# Lipid metabolism in cancer progression and therapeutic strategies

**DOI:** 10.1002/mco2.27

**Published:** 2020-12-24

**Authors:** Yan Fu, Tiantian Zou, Xiaotian Shen, Peter J. Nelson, Jiahui Li, Chao Wu, Jimeng Yang, Yan Zheng, Christiane Bruns, Yue Zhao, Lunxiu Qin, Qiongzhu Dong

**Affiliations:** ^1^ Department of General Surgery, Huashan Hospital & Cancer Metastasis Institute & Institutes of Biomedical Sciences Fudan University Shanghai China; ^2^ Medical Clinic and Policlinic IV Ludwig‐Maximilian‐University (LMU) Munich Germany; ^3^ General, Visceral and Cancer Surgery University Hospital of Cologne Cologne Germany; ^4^ Department of General Surgery, Ruijin Hospital Shanghai Jiao Tong University School of Medicine Shanghai China

**Keywords:** cancer, lipid metabolism, mechanism, microenvironment, therapeutic strategy

## Abstract

Dysregulated lipid metabolism represents an important metabolic alteration in cancer. Fatty acids, cholesterol, and phospholipid are the three most prevalent lipids that act as energy producers, signaling molecules, and source material for the biogenesis of cell membranes. The enhanced synthesis, storage, and uptake of lipids contribute to cancer progression. The rewiring of lipid metabolism in cancer has been linked to the activation of oncogenic signaling pathways and cross talk with the tumor microenvironment. The resulting activity favors the survival and proliferation of tumor cells in the harsh conditions within the tumor. Lipid metabolism also plays a vital role in tumor immunogenicity via effects on the function of the noncancer cells within the tumor microenvironment, especially immune‐associated cells. Targeting altered lipid metabolism pathways has shown potential as a promising anticancer therapy. Here, we review recent evidence implicating the contribution of lipid metabolic reprogramming in cancer to cancer progression, and discuss the molecular mechanisms underlying lipid metabolism rewiring in cancer, and potential therapeutic strategies directed toward lipid metabolism in cancer. This review sheds new light to fully understanding of the role of lipid metabolic reprogramming in the context of cancer and provides valuable clues on therapeutic strategies targeting lipid metabolism in cancer.

## Graphical abstract

Lipid metabolic reprogramming‐mediated crosstalk between cancer cells and tumor microenvironment contributes to cancer progression. Stromal cells, such as adipocyte, CAF, epithelia cells contribute to lipid metabolic reprogramming in cancer cells. Cancer cells undergo lipid metabolism rewiring to generate building materials for membrane, lipid second messenger and energy supply. Cancer cells can also secret lipid metabolites to affect immune cell functions, creating a tumor‐favoring immune microenvironment.

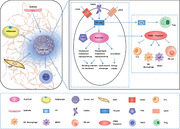

**Abbreviation**
CAF: Cancer associated fibroblast
CTL: cytotoxic T lymphocyte
FA: Fatty acid
FABPs: fatty acid‐binding proteins
FAS: Fatty acid synthesis
LDLR: low‐density lipoprotein receptor
MDSC: myeloid‐derived suppressor cells
NK cell: natural killer cell
PGE2: prostaglandin E2
Th 17: T helper cell 17

## 1 INTRODUCTION

Cancer ranks second in the leading causes of morbidity and mortality worldwide.^1^ Tumor cells possess the advantage of metabolic plasticity that allows them to adapt and survive within harsh microenvironments. Pioneering work in the field of cancer metabolism by Otto Warburg in the 1920s found that cancer cells have a metabolic preference for converting glucose to lactate, even under aerobic conditions, by a process now referred to as the “Warburg effect.^2^” To date, much effort has been made to analyze the metabolic phenotypes that occur in tumors. In the last decade, metabolomics that seeks a detailed profiling of small molecules in a biological sample has emerged as a significant new research tool for characterizing metabolic heterogeneity in cancer.^3^ Through metabolomics, emerging evidence has suggested an important role for metabolic reprogramming in cancer progression, including a deregulated lipid metabolism.^4^

Lipids are represented by a complex group of biomolecules including fatty acids (FAs), glycerides (neutral glycerides and phosphoglycerides), nonglyceride lipids (steroids and sphingolipid), and lipoproteins. Lipids are required for the maintenance of cellular structures, energy supply, and diverse aspects of signal transduction. Amphipathic lipids form the plasma membranes for cellular homeostasis. Changes in lipid metabolism can alter the membrane composition and permeability that is critical for cellular function. Lipid metabolism also generates an array of biological intermediates, many of which can act as signaling molecules that help regulate the diverse set of signaling pathways that control cell growth, proliferation, differentiation, apoptosis, motility, inflammation, survival, and membrane homeostasis.^5^ Lipid metabolism refers to a complex set of molecular processes that include lipid uptake, *de novo* synthesis, transport, and degradation. The dysregulation of lipid metabolism can alter membrane composition, gene expression, signaling pathway activity, and the downstream cellular functions, and thus may directly impact the initiation and progression of diverse disease processes.^6^ The upregulation of lipogenic enzymes has been described in various cancers including colorectal cancer, prostate cancer, ovarian cancer,^7^ gastrointestinal cancer, and lung cancer.^4^ Increased lipid biosynthesis is thought to contribute to tumor cell proliferation in part by providing increased “building materials” for the cell membrane production needed for cell duplication, as well as a supply of energy through β‐oxidation of FAs, and finally, an increase in the “lipid second messenger” molecules that mediate oncogenic pathways. The metabolic reprogramming of lipids can also influence other processes that are critical for cancer progression, such as endoplasmic reticulum (ER) stress and ferroptosis,^8^, ^9^ while inhibition of lipid biosynthesis can help restrict cancer cell survival and tumor growth.

FAs, phospholipids, and cholesterol represent the three major classes of lipids that become dysregulated in tumors. FAs, containing a terminal carboxyl and a hydrocarbon chain varying in carbon lengths and saturation level, represent the initial building materials for lipid synthesis.^4^ Recent reports have highlighted the crucial role that FAs synthesis, uptake, and fatty acid oxidation (FAO) play in cancer development and progression.^10^ Acetyl‐coenzyme A (CoA), the main product of FAO and precursor for lipid biosynthesis, plays a central role in epigenetic regulation by providing the molecular substrates needed for acetylation.^11^ Our group has shown that Acyl‐CoA thioesterase 12 (ACOT12)‐mediated hydrolysis of acetyl‐CoA is associated with hepatocellular carcinoma (HCC) metastasis through the epigenetic upregulation of TWIST2.^12^ Phospholipids and cholesterol are predominant components of cell membranes. These lipids play essential roles in maintaining the malignant cancer phenotype, such as promoting multidrug resistance^13^ and driving distant metastasis^14^ resulting in a poor patient outcome. Recently, the role of several other lipids, such as phosphoinositides, lipoprotein, and triglyceride (TG), has also been elucidated in these settings.^15^ Recent studies have also suggested that lipid metabolism intermediates may serve as metabolic biomarkers for cancer diagnosis and prognosis.^16^ Some genes essential for controlling lipid metabolism in cancer have been suggested as therapeutic targets for cancer treatment. Drugs targeting some of these genes are currently undergoing preclinical investigation including statins that target 3‐hydroxy‐3‐methylglutaryl‐CoA (HMG‐CoA), a rate‐limiting enzyme in cholesterol metabolism.^17^ The general importance of the tumor microenvironment (TME) in promoting and maintaining tumor growth is well recognized. A series of studies have described the impact of lipid metabolism on the TME in cancer progression.^18^ Stromal cells in TME, especially immune cells, undergo lipid metabolic reprogramming to help them survive in the TME. In addition, lipid metabolites present in the TME play a significant role in regulating tumor immunogenicity.^19^ A better general understanding of lipid metabolic reprogramming within the complete cancer environment may provide new prognostic biomarkers and therapeutic targets for cancer treatment.

In the following section, we will discuss recent evidence for lipid metabolic reprogramming by focusing on FA, cholesterol, and phospholipids in cancer progression. We will summarize the diverse regulatory mechanisms underlying lipid metabolic reprogramming in cancer, as well as lipid metabolism‐mediated cross talk between cancer cells and the TME. Finally, we will discuss recent advances in lipid metabolism‐targeting strategies currently in preclinical and clinical development.

## 2 DYSREGULATION OF LIPID METABOLISM IN CANCER

**FIGURE 1:**
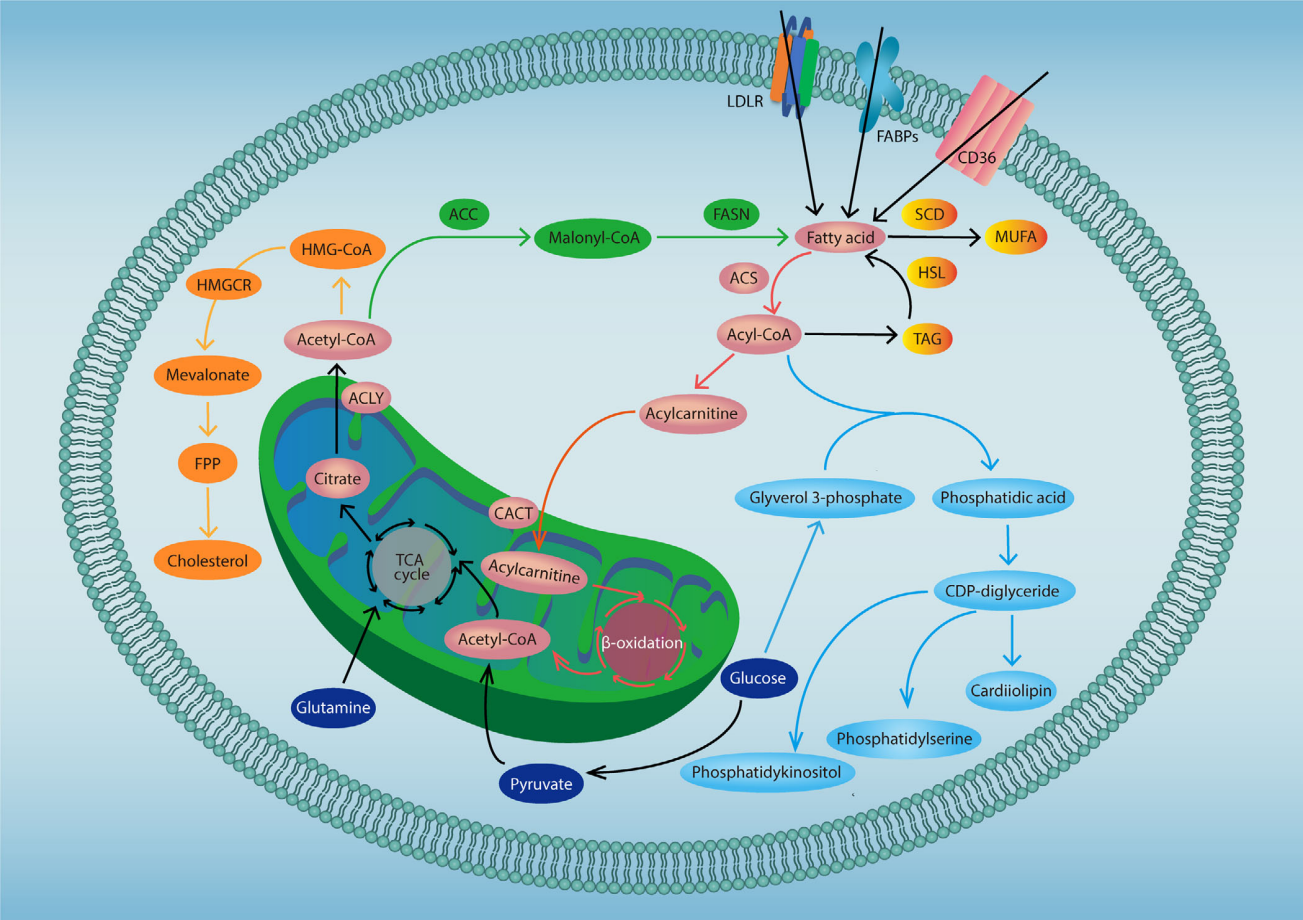
Lipid metabolism in cancer. Glutamine and glucose‐derived acetyl‐CoA are used in the *de novo* synthesis of cholesterol and fatty acids. In addition, exogeneous uptake also contributes to the fatty acid pool in cancer cells. Fatty acids can undergo β‐oxidation to generate ATP and acetyl‐CoA. FA‐derived acyl‐CoA and glucose‐derived glyverol 3‐phosphate serve the main material source for *de novo* synthesis of phospholipids Abbreviations: ACC, acetyl‐CoA carboxylase; ACLY, ATP‐citrate lyase; ACS, acyl‐CoA synthetase; CACT, carnitine acyl‐transferase; CD36, fatty acid translocase/scavenging receptor; FABPs, fatty acid‐binding proteins; FASN, fatty acid synthase; FPP, farnesylpyrophosphate; HMGCR, 3‐hydroxy‐3‐methylglutaryl‐CoA reductase; HSL, hormone‐sensitive lipase; LDLR, low‐density lipoprotein receptor; MUFA, monounsaturated fatty acid; SCD, stearoyl‐CoA desaturase (D9); TAG, triacylglycerol.

Cancer cells are characterized by aggressive proliferation. This requires that the cells develop strategies to acquire nutrients in a microenvironment that is deficient in O_2_ and general nutrient supplies. In addition to an increased demand for glucose, glutamine, and some amino acids, cancer cells undergo lipid metabolic reprograming that allows them to acquire the energy stores and membrane production required for rapid proliferation.^20^ A more comprehensive understanding of how cancer cells utilize lipid metabolic reprogramming to support their malignant phenotype may help identify novel therapeutic targets for cancer treatment (Figure 1).

### 2.1 Reprogrammed FA metabolism in cancer

The relative importance of FAs metabolic reprogramming in cancer has not been recognized until relatively recently. FAs play an important role in the synthesis of diverse lipid molecules, including TGs, phospholipids, sphingolipids, and sterols. Normal mammalian cells obtain FAs largely by exogenous uptake, whereas the principle source of FAs in cancer cells derives largely from *de novo* synthesis rather than the microenvironment. Metabolomics profiling has revealed enhanced FA synthesis and palmitoleic acid generation in cancer, which play essential roles in cancer growth.^21^ The fatty acid synthase (FASN)‐encoded gene is highly expressed in several human cancers, highlighting the abnormal activation of *de novo* FA synthesis pathway. FASN has been subsequently reported to promote angiogenesis in colorectal cancer.^22^ In pancreatic cancer, increased FASN was linked to disease progression and poor survival, as well as gemcitabine resistance through enhanced ER stress.^23^

FAs can be subgrouped into saturated and unsaturated molecules. The ratio of saturated to unsaturated FAs is crucial for protecting the cell from lipotoxicity. Stearoyl‐CoA desaturase (SCD) is an integral membrane protein found in the ER that catalyzes the rate limiting step in formation of the monounsaturated FAs oleic acid (18:1) or palmitoleic acid (16:1) from stearoyl‐(18:0) or palmitoyl‐CoA (16:0). Several studies have reported that SCD is significantly increased in tumors, and SCD‐mediated desaturation of FA may represent important step for cell survival as the accumulation of saturated FA leads to lipotoxicity and ER stress.^8^ Inhibitors targeting SCD have been shown to induce the apoptosis of tumor cells and to inhibit tumor formation in a xenograft model of gastric cancer.^24^ In collaboration with the Wang Group at National Institutes of Health (NIH), our group has used liver cancer tissues paired with adjacent normal tissues for detailed metabolomics analysis and general screening for differentially expressed genes. We were able to identify 28 altered metabolites and 169 differentially expressed genes associated with HCC progression. Further analysis showed that an SCD metabolic pathway signature was significantly correlated with progression of HCC. Functional studies subsequently showed that interference with expression of SCD could reduce the migration and invasion of HCC.^25^ The central role of *de novo* FA synthesis activation in cancer is well recognized; however, the potential contribution of FA uptake from TME is less appreciated. Recent reports have shown that tumors also absorb FAs from the tumor environment, suggesting that FA uptake may be as important as *de novo* synthesis to tumor progression. Specific molecular transporters, such as FA translocase (FAT/CD36), the FA transport proteins (FATPs/SLC27A), low‐density lipoprotein receptor (LDLR) and FA binding proteins (FABPs), are needed for FA uptake. Human oral cancer metastasis initiating cells have been shown to express the surface protein CD36 that helps promote the metastasis of oral cancer through CD36‐mediated FAs uptake.^26^, ^27^ FATP2, a member of the FATP family, is upregulated in polymorphonuclear myeloid‐derived suppressor cells (PMN‐MDSCs), a group of pathologically activated neutrophils shown to contribute to treatment failure and predict poor disease prognosis.^28^, ^29^ In glioblastoma (GBM), epithelial growth factor receptor (EGFR) vIII‐activated, PI3K/sterol regulatory element‐binding protein (SREBP) 1‐dependent LDLR upregulation was shown to promote GBM cell survival, while targeting LDLR using nuclear liver‐X‐receptor (LXR) agonists has shown therapeutic value for GBM patients.^30^ Work by Li et al has shown that the transmembrane glycoprotein CD147 is highly expressed in various tumors, and is also an important regulator of FA metabolism.^29^

In summary, FAs are involved in diverse biological processes linked to tumor growth. FAs are needed for phospholipids, the structural molecules of cell membranes. To meet the increasing demand during progression, tumor cells absorb or synthesize a large amount of FAs. Specific types of tumors (such as prostate cancer) use β‐oxidation of FA as their main source of energy.^31^ FAs are also important in the production of lipid‐based signaling molecules including inositol phosphate, lysophosphatidic acid (LPA), and the prostaglandins.^32^ Recent studies have reported that the expression of key genes linked to the FA metabolism pathway is increased. The suppression of these metabolic enzymes by specific inhibitors can inhibit tumor growth.^33^

### 2.2 Reprogrammed phospholipid metabolism in cancer

Lipids containing phosphoric acid are referred as phospholipids. Phospholipid is an essential component of the cell membrane and is important in maintaining the structure and normal function of cell membrane, as well as regulating signaling transduction and the cell cycle. Using acyl‐CoAs as donors, phospholipids are formed by a *de novo* pathway referred to as the Kennedy pathway. They are then modified by subsequent cycles of deacylation and reacylation through a remodeling pathway known as the Lands' cycle.^34^ Phospholipid content has been shown to regulate various carcinogenic processes such as tumor growth, migration, and metastasis. Phosphoglycerides and sphingolipids are two major phospholipid structures. In addition, phospholipid metabolites, such as the platelet activating factor (PAF), arachidonic acid (AA), diacylglycerol (DG), and creatine triphosphate (IP3), are also critical for the biologic function of cells.

#### 2.2.1 Phosphoglyceride metabolism and cancer

Phosphoglyceride, also called glyceropholipids, is the most abundant phospholipids and the major constituents of membrane bilayers. In phosphoglycerides, two FAs are esterified to a glycerol backbone. They include various head groups, such as choline, ethanolamine, serine, glycerol, or inositol. Phosphoglycerides can be further divided into phosphatidylcholine (PC), phosphatidylserine (PS), phosphatidylethanolamine (PE), phosphatidylglycerol (PG), phosphatidylinositol (PI), and cardiolipin.^13^ The *de novo* synthesis of phosphoglycerides is achieved through the CDP‐choline pathway (or Kennedy pathway), where three enzymes, choline kinase, phosphocholine cytidylyltransferase (CCT), and 1,2‐diacylglycerol cholinephosphotransferase, contribute to the biosynthesis. The changes in phosphoglyceride metabolism that occur in cancer can be attributed to dysregulation of the phosphoglyceride metabolism‐related rate‐limiting enzymes and are critical for tumor growth. For example, the activity of phospholipase A1/A2 (PLA1/2), which can generate free FAs, and 2‐acyl lysophospholipid or 1‐acyl lysophospholipid through modifying the sn‐1 and sn‐2 positions of the glycerol moieties of phospholipids, is significantly increased in cancer cells.^35^ Secreted PLA2 can also promote the proliferation of astrocytoma driven through EGFR signaling. In stage II colorectal cancer, patients with PLA2‐negative tumors show significantly longer disease‐free survival time, suggesting that PLA2 can be used as a prognostic predictor for colorectal cancer.^36^ Phospholipase D (PLD) hydrolyzes PC to yield phosphatidic acid (PA) and free choline, and has been implicated in the development and progression of cancer.^37^ PLD has a direct role in cancer cell proliferation, migration, invasion, metastasis, and tumor angiogenesis.^38^ In colorectal cancer, inhibition of PLD activity was shown to suppress the generation of PA, and to inactivate mTOR signaling, thereby interfering with the proliferation of cancer cells.^39^ In addition, several other enzymes implicated in phospholipid metabolism have also been reported to play critical roles in tumor progression, including Lipins^40^ and LPCATs.^41^

Phosphoglycerides and its derivatives are important to the maintenance of some malignant cancer phenotypes, and have been linked to a poor cancer prognosis. PC is the most predominant phospholipid in eukaryotic cells and is enriched in human colorectal cancer where it can serve as a diagnosis marker.^41^ The expression of phosphocholine CCT, an important enzyme in PC synthesis, is also of prognostic value in cancer.^42^ PI is a rather minor membrane component when compared to the other phospholipids; however, PI and the closely related PI phosphates (PIPs) such as PI 4‐phosphate (PI4P), PI 4,5‐bisphosphate (PI‐4,5‐P2), and inositol 1,4,5‐triphosphate (IP3), are important in hormone and growth factor signaling, and have been linked to aberrant signaling processes in cancer.^43^ PC and PI are important sources of the secondary messenger DG that is generated from the respective phospholipid by phospholipid‐specific phospholipase C (PLC). PLC is essential to tumor progression and has been identified as potential cancer treatment target.^44^ LPA, a phospholipid hydrolysate, is a bioactive lysophospholipid that can regulate diverse biological process by binding to specific receptors. Extracellular LPA is generated by the enzyme autotaxin/ectonucleotide pyrophosphatase phosphodiesterase 2 that contains a lysophospholipase D domain and can cleave lysophospholipids into LPA, or it can be generated by secreted phospholipases A1 and A2 that cleave the FAs chains from glycerophospholipids. Intracellular LPA is generated by intracellular phospholipases A1 and A2, glycerol 3‐phosphate acyltransferase, or monoacylglycerol kinase.^45^ A role for autoxin and phospholipases A1/A2 in cancer progression has been suggested.^46^, ^47^ Analysis of clinical data has indicated an increased level of LPA in cancer, suggesting that LPA may serve as a diagnosis or prognosis biomarker for cancer.^48^, ^49^ Emerging studies have suggested that LPA is involved in central aspects of tumor activities, including activation of proliferation signaling, unlimited cell growth and resistance to apoptosis, angiogenesis and lymphangiogenesis, epithelial to mesenchymal transition (EMT)‐mediated tumor invasion and metastasis, genome instability, tumor‐promoting inflammation, metabolic reprogramming, and anticancer immune evasion. AA is an important phospholipid metabolite that is associated with carcinogenesis. AA can promote tumor cell growth via activation of PLC and PKC that, in turn, regulates intracellular Ca and cAMP concentrations, and enhanced oxygen consumption and CO_2_ production. In addition, AA also contributes to tumor progression through the generation of biologically active metabolites, such as PGG2, leukotriens (LTs), and eicosanoids, which are produced through three different enzymatic pathways—cyclooxygenase (COX), lipoxygenase (LOX), and cytochrome P450 (CYP) pathways, respectively.^50^ AA‐derived PGE2 also plays a vital role in tumor progression via its interaction with four distinct E‐prostanoid receptors (EP1‐4).^51^ In addition, PGE2 can also promote tumor development through suppression of tumor immunity. 5‐LOX, an important LOX isoform, is involved in the progression of various cancers, such as breast, pancreatic, lung, prostate, liver, and intestine cancer.^52^ The molecular product of 5‐LOX activity, LTB4, can influence carcinogenesis via regulation of the tumor cell phenotype, as well as by reshaping the microenvironment to favor cancer progression.^53^

#### 2.2.2 Sphingolipid metabolism and cancer

Sphingolipids are structural components of cell membranes that play important roles in maintaining membrane function and integrity. In addition to acting as structural molecules, sphingolipids are represented by a large family of bioactive lipids that function as intracellular and extracellular mediators that have critical roles in the regulation of diverse cellular processes such as cell proliferation, apoptosis, senescence, and transformation.^54^ Sphingolipids such as the ceramides and sphingosine‐1‐phosphate (S1P) have been implicated as important modulators in cancer growth, progression, and chemotherapy. Ceramides and S1P can exert opposing functional roles for determining cell fate in response to specific stimuli. Ceramides are involved in mediating cell‐stress responses such as cell cycle arrest and apoptosis, whereas S1P has been shown to stimulate cell survival, proliferation, and migration.^55^ These sphingolipids with apparently opposing biologic actions can be interconverted within cells, suggesting that the balance among them may be critical for cell fate.

Bioactive sphingolipids are regulated by a series of enzymes and metabolites. Approximately 40 enzymes in mammals are involved in their metabolism.^56^ The level of sphingolipid molecules is regulated by metabolic enzymes, and changes in their expression or activity have effects on the induction of cancer, cell survival, and death.^54^ Serine palmitoyltransferase (SPT) initiates sphingolipid synthesis by acting on serine and palmitoyl CoA to produce 3‐ketosphinganine in the ER. As the first rate‐limiting enzyme in sphingolipid metabolism, the enzymatic activity of SPT affects the tumorigenesis of many cancers.^57^ SPT inhibition through myriocin can prevent the proliferation of melanoma cells and induce cell cycle arrest in the G(2)/M phase.

Ceramides are key intermediates in sphingolipid metabolic pathways in the generation of complex sphingolipids. The synthesis and/or accumulation of ceramides in response to individual types of stress stimuli can act as enhancers of apoptotic programs through the induction of senescence, autophagy, and ER stress.^58^ However, many tumors show enhanced ceramide metabolism and increased activities of relevant enzymes including glucosylceramide synthase (GCS), sphingomyelin synthase (SMS), ceramide kinase (CERK), acid ceramidase (AC), and/or sphingosine kinase (SPHK), which increases generation of sphingolipids resulting in enhanced prosurvival functions.^59^ GCS is overexpressed in drug‐resistant breast, ovary, cervical, and colon cancer cells, and modulates drug resistance in these cancers via upregulation of MDR1 expression.^60^ In human glioma, nonsmall lung adenocarcinoma, and leukemia, activation of SMS has been shown to trigger cell cycle arrest, cell differentiation, and autophagy or apoptosis.^61^ CERK is required for mammary tumor recurrence following HER2/neu pathway inhibition. Elevated CERK expression is associated with an increased risk of recurrence in women with breast cancer.^62^ CERK inhibitor decreases the number of cells in S phase and induces M phase arrest in breast and lung cancer cell lines, which supports a proliferative role for CERK in these cancers.^63^ Ceramides with specific fatty acyl chains produced through CerS have unique roles in cancer cell death and survival.^64^ For example, a tumor‐suppressive role of CerS1 has been identified in the pathogenesis of head and neck squamous cell carcinoma (HNSCC)^65^ and is found in some lung adenocarcinoma cell lines . By contrast, enhanced levels of CerS1 mRNA in breast cancer tissues have been correlated with poor prognosis.^66^ In human nonsmall‐cell lung cancer (NSCLC) cells and tumors, enhanced expression of CerS6 was observed and associated with increased tumor invasiveness, metastasis, and poor patient survival.^67^ However, CerS6 overexpression was linked to reduced plasma membrane fluidity and reduced cell migration in human breast cancer cells.^68^ These studies suggest that CerSs play different, even opposing roles in the development of different cancers.

S1P is an additional important bioactive lipid in the sphingolipid family. It is produced through sphingosine kinase (SKs)‐mediated phosphorylation of sphingosine, which is a catalytic product of ceramide modification by ceramidase.^69^ It has been suggested that the balance between levels of ceramide and S1P plays an important role in determining cell survival and apoptosis. The regulatory effect of S1P in cell physiology and pathology is driven through their binding to G protein‐coupled S1P receptors (S1PRs) leading to the activation of downstream signaling pathways including Akt and ERK1/2.^70^ In addition, intracellular S1P can epigenetically regulate gene expression.^71^ S1P concentrations have been shown to be increased in cancer interstitial fluid as compared to normal tissue in both experimental cancer models and in cancer patients.^72^ Emerging evidence has revealed a role for S1P in cancer progression via signaling through S1PR1‐5. S1PR1‐5 are largely regarded as protumorigenic receptors as they can activate numerous tumor‐associated signaling pathways, such as the Ras/ERK, PI3K/Akt, STAT3, and PLC pathways.^73^ In this way, S1P can be seen to promote tumor progression via facilitating important properties of cancer cells, such as cancer cell growth, cell transformation, EMT, enhancing tumor‐promoting inflammation, and promoting angiogenesis^74^, ^75^ . The SK1 and SK2 enzymes catalyze substrates for the production of S1P. A series of studies have shown an enhanced expression of SK1 and SK2 in several types of cancer cells, and have shown an association between high SK1/2 expression and a poor prognosis in cancer.^76^ Experimental treatment with anti‐S1P monoclonal antibody was shown to reduce tumor progression in murine xenografts, suggesting that the lipid S1P may also represent a target for cancer therapy.^77^

### 2.3 Reprogrammed cholesterol metabolism in cancer

Epidemiological evidence has shown a correlation between high cholesterol diet and an increased risk of cancer, suggesting a potential contribution of cholesterol to cancer initiation and progression.^78^ Recently, cholesterol metabolic reprogramming in cancer has gained attention based in part on its important role in maintaining cellular homeostasis, such as providing essential hormones, including vitamin D, progesterone, and estrogens; as well as its role in forming lipid rafts, a vital cell membrane structure in cancer cells.^79^ Many tumor‐relevant proteins are located in lipid rafts, such as the death receptors, protein kinases, and calcium channels, making lipid rafts an important platform for oncogenic signaling pathways. Cholesterol deficiency in cell membranes has been shown to inhibit cancer progression.^80^ Numerous metabolites generated by *de novo* modification of cholesterol, such as mevalonic acid, farnesyl pyrophosphate (FPP), and geranylgeranyl pyrophosphate, are also important for cancer cell growth, based, in part, on the critical roles they play in oncogenic signaling pathways, such as the Hedgehog, PI3K/Akt/mTORC1, and Wnt/β‐catenin pathways.^81^, ^82^, ^83^ *De novo* synthesis of cholesterol relies mainly on the mevalonate pathway and utilizes acetyl‐CoA. Increased consumption of glucose, glutamine, and acetate in cancer cells leads to an enhanced production of acetyl‐CoA,^84^, ^85^ which can be produced through the mevalonate pathway to form HMG‐CoA . Depending on 3‐hydroxy‐3‐methylglutaryl‐CoA reductase (HMGCR), HMG‐CoA can be reduced to form mevalonate, and undergo subsequent modification to form the sterols and isoprenoids that are essential for tumor growth.^86^

Cholesterol is commonly stored in the cytosol in the form of lipid droplets after esterification by acyl‐CoA cholesterol acyl transferase (ACAT). The level of cholesterol in the cell is tightly controlled through *de novo* synthesis and exogenous uptake.

Lipid metabolism and homeostasis is transcriptionally regulated through two classes of transcriptional factors, the SREBPs and LXRs. SREBPs, a family of helix‐loop‐helix leucine zipper (HLH‐LZ) transcription factors, were first identified by Brown et al as important regulators of cholesterol homeostasis by regulating genes involved in the mevalonate and LDLR pathways.^87^ In mammals, SREBPs can be divided into three isoforms, SREBP1a and SREBP1c encoded by sterol regulatory element binding transcription factor 1 (SREBF1), and SREBP2 encoded by the sterol regulatory element binding transcription factor 2 (SREBF2). INSIG proteins, anchor proteins of the ER, can bind to the SREBP cleavage activating protein (SCAP) that results in retention of the SREBPs/SCAP complex in the ER. When cellular sterol concentrations decrease, inactive precursors of SREBPs are escorted by SCAP to the Golgi where they are processed into mature SREBPs, which translocate to the nucleus and bind to E‐boxes or sterol regulatory elements (SREs) in the promoter regions of their target genes.^88^ SREBP1 mainly regulates FAs synthesis‐associated genes, whereas SREBP2 regulates genes involved in cholesterol biosynthesis, based in large part by their different affinity to specific SREs.^89^ In the nucleus, SREBP activity is influenced by their interaction with other transcription factors, such as hepatocyte nuclear factor 4 (HNF4) and LXR.^90^ The LXR transcriptional factor is critical in control of cholesterol homeostasis. When intracellular cholesterol content is increased, LXRs are activated to help drive cholesterol efflux by inducing the transcription of genes linked to the efflux of cholesterol, such as ATP binding cassette subfamily A member 1 (ABCA1) and ATP binding cassette subfamily G member 1 (ABCG1).^91^ LXR can also bind to SREBP to reduce its transcriptional activity, thus reducing *de novo* synthesis of cholesterol.^92^

Cancer cells often show an increased activity of SREBPs, leading to an increased expression of enzymes involved in the mevalonate pathway, including HMGCR, a rate‐limiting enzyme in the mevalonate pathway.^86^ An increased activity of SREBPs in tumors is linked to oncogenic signaling activation, a mechanism that is independent of sterol abundance.^93^ In addition to dysregulation of the *de novo* synthesis of cholesterol, altered cholesterol influx mediated by factors such as CD36, VLDLR, LDLR, the scavenger receptor class B member 1 (SCARB‐1), ABCG1, and ABCA1,^94^ also contributes to cholesterol homeostasis in cancer cells through a process largely transcriptionally regulated by LXR.^95^

Cancer cells undergoing cholesterol metabolic reprogramming show an enhanced malignant phenotype that includes enhanced drug resistance,^13^ increased proliferation, immune escape, metastasis, and the presence of “stemness” properties.^96^ Cholesterol metabolic reprogramming is strongly linked to tumor growth. Inhibiting cholesterol production affects the fluidity and overall lipid raft formation, thus potentially modifying the normal function of cell membrane.^97^ CD36 is an important lipid transporter that plays a critical role in cancer cell growth and metastasis via transport of lipid into cells, and by inhibition of CD36 that can significantly reduce metastatic activity.^26^, ^98^ Furthermore, inhibiting the production of intermediates of the mevalonate pathway that critical for cancer cell growth, such as FPP and gernaylgerany parophosphate (GGPP), can also promote the apoptosis of cancer cells.^99^ Intracellular cholesterol‐mediated regulation of cell apoptosis, and cell cycle progression may be partially attributed to cholesterol‐enriched lipid rafts that are associated with proteins engaged in the regulation of pro‐oncogenic and apoptosis pathways.^100^ It can thus be seen that cholesterol metabolism plays a significant role in cancer cell survival and progression.

## 3 MOLECULAR MECHANISMS OF LIPID METABOLIC REPROGRAMMING

Dysregulated lipid metabolism is an important aspect in the metabolic rewiring that occurs during cancer transformation and promotes cancer survival and progression. Better insight into this biology should identify new therapeutic targets for cancer intervention.

### 3.1 Transcription factors contributing to lipid metabolism reprogramming

As detailed earlier, LXR and the SREBPs are the transcription factors that contribute to lipid metabolism regulation, lipid uptake, transport, efflux, and the *de novo* synthesis of lipids.^101^ Recent reports have suggested a regulatory role for additional transcriptional factors in the dysregulation of lipid metabolism seen in tumor cells. NANOG, a transcription factor activated during embryonic development and cellular reprogramming,^102^ is also linked to stemness and is involved in the reprogramming of lipid metabolism. Nanog‐stimulated FAO driven through transcriptional activation of FAO‐associated genes, such as acyl‐CoA dehydrogenase, and enhanced lipid desaturation through upregulation of SCD1 have been shown to contribute to enhanced self‐renew and drug resistance in liver cancer cells.^103^ RUNX1 belongs to a family of transcriptional factors that orchestrate diverse cellular activities including cellular proliferation and differentiation.^104^ A recent study found that RUNX1 can modulate membrane organization by influencing FA production by direct transcriptional regulation of SCD1 and sterol O‐acyltransferase 1, two important enzymes involved in lipid metabolism. This can further facilitate the signal transduction required for the rapid proliferation of cancer cells.^105^ MYC is an important protooncogenic transcriptional factor that contributes to cellular growth, cell transformation, and tumorigenesis.^106^ The role of MYC in lipid metabolism is well recognized. MYC has been shown to increase mitochondrial synthesis of acetyl‐CoA and to induce the *de novo* synthesis of the FA palmitate. Recent work has also suggested that MYC may contribute to the *de novo* synthesis of FA by activating FASN and SCD.^107^ In addition, MYC also plays a role in the regulation of FAO. MYC inhibition leads to decreased levels of FAO through inhibition of the mitochondrial respiratory complex and β‐oxidation in neuroblastoma cells, which results in growth inhibition and cell apoptosis.^108^ The same effect has also been observed in hepatoblastoma cells.^109^ FAO inhibition can reduce the energy metabolism and suppress tumorigenesis in MYC‐high expressed triple negative breast cancer (TNBC), suggesting that MYC could serve as a biomarker for determining which patients may benefit from FAO inhibition therapy.^110^ Hypoxia inducible factors (HIFs) are critical mediators of hypoxic stress in tumors and are associated with diverse aspects of cellular activity, such as proliferation, differentiation, migration, and chemoradiotherapy resistance.^111^ HIF‐2α, a member of the HIF family, plays a critical role in proliferation, angiogenesis and stemness properties of colorectal cancer, pancreatic cancer, and ovarian cancer.^112^ Qiu et al reported that HIF‐2α can regulate lipid storage, thus impacting ER homeostasis in clear cell renal cell carcinoma (ccRCC).^113^ An increased expression of HIF‐2α in tumor cells may lead to enhanced expression of the LD coat protein gene PLIN2. This protein can increase lipid synthesis and storage in the liver and is commonly used as a marker for cellular lipid accumulation.^114^ HIF‐2α‐mediated expression of PLIN2 can help derive the lipid storage required for ER homeostasis and contribute to tumor growth and pharmacologic resistance to ER stress.^113^

### 3.2 Signaling pathways contributing to lipid metabolism reprogramming

**FIGURE 2:**
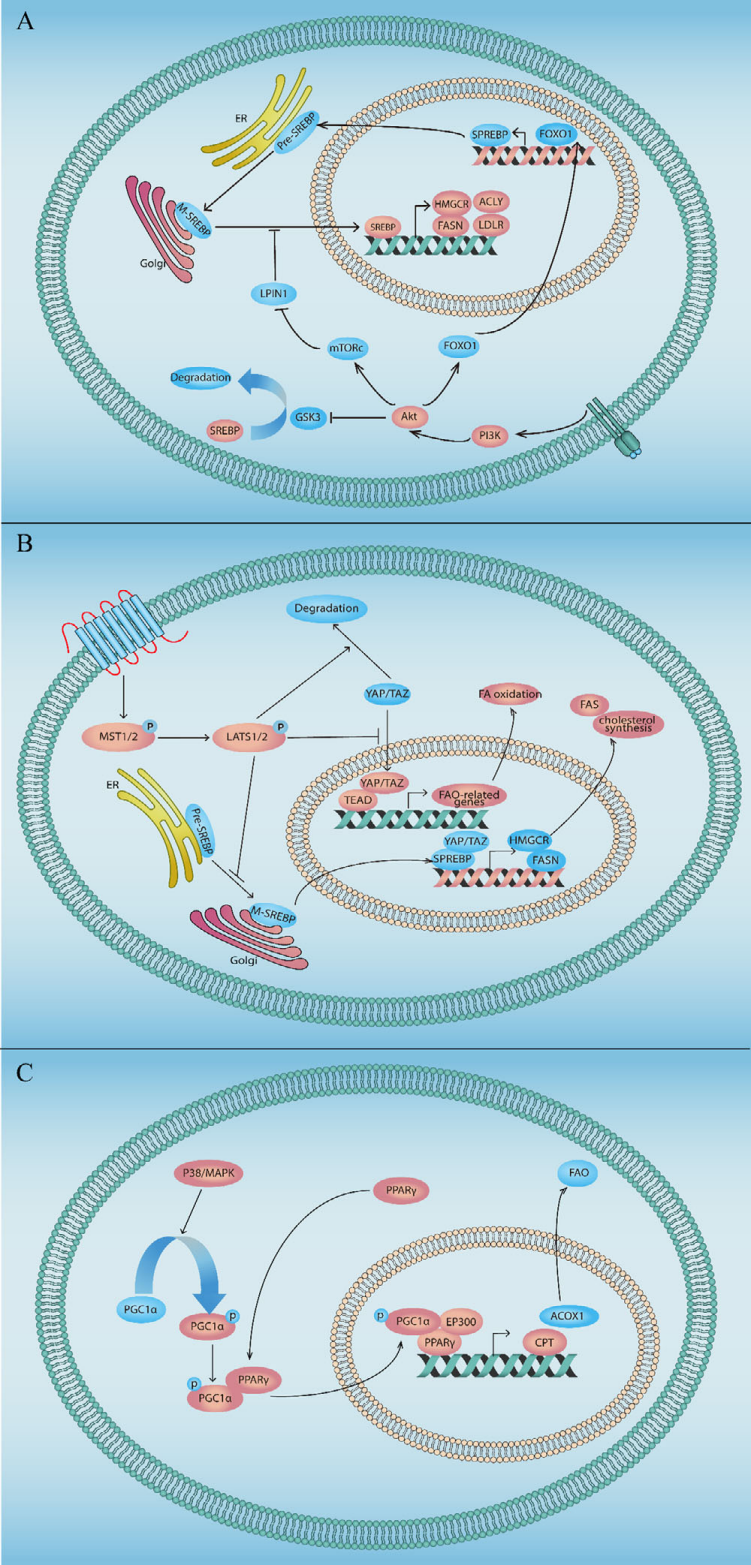
Signaling pathways in lipid metabolic reprogramming. (A) The PI3K/Akt signaling pathway regulates lipid metabolism in cancer cells. PI3K/Akt can increase expression of FOXO1 leading to initiation of SREBP transcription. mTORC, a downstream target of PI3K/Akt signaling, can inhibit activation of LPIN, which will otherwise sequester SREBP and inhibit its translocation into nucleus. In addition, Akt can inhibit the activity of GSK3 to inhibit degradation of SREBP. (B) The Hippo signaling pathway regulates lipid metabolism in cancer cells. Noncannonical Hippo pathway regulates lipid metabolism through inhibiting the maturation of SREBP via preventing its translocation from ER to Golgi. LATS1/2 also promotes YAP/TAZ degradation, which will otherwise initiate the expression of FAO‐related genes to promote FA oxidation in cancer cells. In addition, YAP can also interact with SREBP in the nucleus to enhance its transcriptional activity and increasing expression of downstream targets such as HMGCR and fatty acids synthase. (C) p38 MAPK participates in the regulation of lipid metabolism via direct phosphorylation of PGC‐1α to facilitate recruitment of the coactivator to PPARγ target genes and driving chromatin remodeling, promoting PPARγ‐dependent gene transcription Abbreviations: ACLY, ATP‐citrate lyase; ACOX1, acyl‐CoA oxidase 1; CPT, carnitine palmitoyltransferase; EGFR, epidermal growth factor receptor; ER, endoplasmic reticulum; EP300, E1A binding protein p300; FAS, fatty acid synthesis; FASN, fatty acid synthase; FOXO1, forkhead box O1; GSK3, glycogen synthase kinase 3; HMGCR, 3‐hydroxy‐3‐methylglutaryl‐CoA reductase; HSL, hormone‐sensitive lipase; LATS1/2, large tumor suppressor kinase 1/2; LDLR, low‐density lipoprotein receptor; M‐ SREBP, mature SREBP; MST1/2, macrophage stimulating 1/2; mTOR, mammalian target of rapamycin; p38 MAPK, p38 Mitogen‐activated protein kinase; PPARγ, peroxisome proliferator activated receptor gamma; PGC‐1α, PPARG coactivator 1 alpha; Pre‐SREBP, premature SREBP; SREBP, sterol regulatory element binding protein; TEAD, TEA domain transcription factor; YAP/TAZ, Yes1‐associated transcriptional regulator/Tafazzin.

Cancer cells typically show an overactivation of oncogenic signaling pathways. Indeed, an aberrant signaling of key pathways has been associated with lipid metabolic reprogramming in different cancer types (Figure 2).

#### 3.2.1 The NF‐κB signaling pathway

NF‐κB, a key transcriptional factor involved in regulaton of the inflammatory response, is also an important survival signaling pathway found to be over‐activated in many cancers.^115^ NF‐κB signaling can also influence lipid metabolic reprogramming. Inhibition of NF‐κB can lead to decreased lipid accumulation in cells, suggesting an essential role for NF‐κB in lipid metabolism.^116^ The SCD1 enzyme is responsible for converting saturated FAs to ∆9‐monounsaturated FAs.^117^ Li et al reported that SCD1‐mediated lipid desaturation plays an essential role in maintaining ovarian cancer stemness properties that is mediated through NF‐κB signaling. NF‐κB can directly bind to the promoter of SCD1 to initiate transcription, effectively forming a positive feedback loop contributing to the phenomenon of cancer stemness.^118^

#### 3.2.2 The PI3K/Akt signaling pathway

The PI3K/Akt pathway is often overactivated in cancer cells resulting in enhanced cell growth and proliferation.^119^ Several studies have found that the PI3K/Akt pathway can also influence lipid metabolism reprogramming in cancer cells. Akt signaling is reported to prevent SREBP1 degradation and thereby promote lipid *de novo* synthesis. Glycogen synthase kinase 3 (GSK3) phosphorylates SREBP1 (Ser‐434 in SREBP1) leading to the degradation of SREBP1 in the nucleus. Akt can also inhibit GSK3 and thereby prevent degradation of mature SREBP1.^120^ In addition, PI3K/Akt/mTORC can also enhance lipid synthesis and promote cell growth via stimulation of SREBP1 accumulation within the nucleus.^121^ EGFR is an oncogene that belongs to the receptor tyrosine kinase family. EGFR activation can promote cell proliferation via activation of the PI3K/Akt signaling pathway. EGFRvIII‐positive GBM shows increased lipogenesis in response to the PI3K/Akt signaling pathway‐dependent overactivation of SREBP1 that, in turn, is linked to tumor growth.^122^ Subsequently, it has been found that SREBP1 overactivation, caused by the EGFRvIII/PI3K/Akt‐dependent pathway, promotes LDLR expression and an increase in cholesterol uptake needed for survival of GBM.^30^ The PI3K pathway activity can also be influenced by a reduction in intracellular levels of PIP3 induced by PTEN, a dual phosphatase with both protein and lipid phosphatase activities, which exerts a significant effect on cell survival, proliferation, differentiation, and metastasis.^123^ Recently, a regulatory role for the PTEN/PI3K/Akt signaling pathway in lipid metabolic reprogramming was identified and was shown to control SREBP expression via transcriptional and posttranscriptional events. Several downstream targets of the PI3K/Akt signaling pathway including the forkhead box O1 (FOXO1) transcription factor can transcriptionally regulate lipid metabolism by modulating SREBPs expression.^124^ PI3K/Akt is also involved in the transport of SREBP2 from the ER to the Golgi, thus contributing to SREBP2 maturation and activation. A recent study has shown that activated Akt can phosphorylate cytosolic phosphoenolpyruvate carboxykinase 1 (PCK1), that then translocaes to the ER where the phosphorylated PCK1 can bind to, and phosphorylate, INSIG1/2 leading to enhanced lipogenesis in HCC.^93^

#### 3.2.3 The Hippo signaling pathway

The Hippo signaling pathway is an important regulator of cell proliferation and differentiation. Dysfunction of Hippo pathway signaling leads to constitutive activation of the Yes1‐associated transcriptional regulator (YAP) and Tafazzin (TAZ) resulting in the regulation of key downstream target genes that ultimately contribute to the proliferation of cancer cells.^125^ ^,^ YAP/TAZ has been reported to interact with mature SREBPs within the nucleus leading to enhanced transcriptional activities and increased expression of downstream targets, such as HMGCR and the fatty acids synthase (FAS). Activation of the Hippo signaling pathway was found to reduce hepatic steatosis in diabetic mice.^126^ YAP also appears to participate in regulating FAO, and thus in helping cancer cells undergoing metastasis via the tumor‐draining lymph nodes (LNs) to survive within the LN microenvironment.^127^ Interestingly, this process was shown to be driven by CYP7A1‐mediated conversion of cholesterol into bile acids, which was previously reported to act as a signal to activate YAP.^128^ In addition to canonical Hippo pathway signaling, noncanonical Hippo pathway signaling can also contribute to lipid metabolic reprogramming. LATS2 can regulate lipid metabolism in a YAP/TAZ‐independent manner by interacting with precursors of SREBPs when they are located within the ER, and thus, inhibit the maturation of SREBPs.^129^ In mouse liver, a tissue‐specific knockout of LATS2 showed excessive hepatic cholesterol accumulation with eventual development of fatty liver disease. These animals were effectively deprived of their ability to recover from high cholesterol‐induced liver damage, and eventually developed premalignant pathology such as ductal reaction and a hyperproliferation of oval cells.^129^

#### 3.2.4 The MAPK signaling pathway

The MAPK signaling pathway is important in intra‐ and intercellular communication, and participates in the regulation of basic cell functions, such as proliferation, differentiation, and survival.^130^ Evidence suggests that the MAPK signaling pathway is also involved in control of lipid metabolism following the observation that p38α inhibition impairs cholesterol homeostasis via inactivation of SREBP1, leading to restricted cancer cell proliferation in liver and prostate cancer.^131^ U0126‐based MAPK inhibition resulted in decreased nuclear accumulation of SREBP1/SREBP2, and in reduced expression of downstream target genes, leading to suppression of the metastatic progression of prostate cancer.^132^ Several studies have shown that the regulatory role of MAPK in lipid metabolism acts via regulation of peroxisome proliferator activated receptor gamma (PPARγ) activity. p38 MAPK can influence the regulation of lipid metabolism via direct phosphorylation of the PPARG coactivator 1 alpha (PGC‐1α) and E1A binding protein p300 (EP300) that facilitates the recruitment of the coactivator to PPARγ target genes, and influences chromatin remodeling leading to PPARγ‐driven target gene transcription. In liver cancer, the MAPK/PPARγ axis has been shown to contribute to CD147‐mediated FA metabolic reprogramming and to play a role in cancer cell proliferation and metastasis.^133^

#### 3.2.5 The Wnt/β‐catenin signaling pathway

Wnt/β‐catenin signaling is an important pathway that controls cell fate and stemness properties during cancer progression.^134^ Recent studies have revealed that the Wnt/β‐catenin pathway can also exert the opposite effect on lipid metabolism, as described above for proliferator‐activated receptor alpha (PPARα) for FA transport, and mitochondrial β‐oxidation.^135^ Senni et al reported that β‐catenin plays a pivotal role in determining which kind of energy source a cell uses to support growth by negatively regulating PPARα.^136^ In breast cancer, knockdown of β‐catenin led to increased expression of acetyl‐CoA carboxylase (ACC) and FASN, proteins involved in the *de novo* synthesis of FAs. In addition to increased expression of ACC and FASN, β‐catenin is also linked to mitochondrial dysfunction which could not be offset by an increased *de novo* synthesis of lipid in tumor cells.^137^ However, tissue metabolomics in HCC have suggested that the presence of a CTNNB1 mutation‐mediated activation of the Wnt/β‐catenin pathway did not lead to metabolic remodeling.^138^ Wnt/β‐catenin pathway signaling is complex and highly context dependent. The regulatory role of Wnt/β‐catenin in lipid metabolism requires further study. It will be important to determine whether Wnt/β‐catenin regulates lipid metabolism in a tissue‐specific manner, and what potential contribution of β‐catenin‐mediated lipid metabolism may play in lipid rewiring in cancer. When considered the central role of Wnt/β‐catenin signaling in cell differentiation, it will also be interesting to determine if β‐catenin‐mediated lipid metabolism rewiring is essential for developmental and cellular differentiation.

#### 3.2.6 The AMPK signaling pathway

As a central regulator of cellular metabolism, the protein kinase AMPK is a highly conserved sensor of intracellular adenosine nucleotide levels, which becomes activated when intracellular ATP production falls.^139^ With a decrease in cellular energy level, the subsequent activation of this enzyme can stimulate FA oxidation leading to enhanced ATP via the activation of downstream target enzymes involved in lipid metabolism, such as acetyl‐CoA carboxylase (ACC1 and ACC2) and HMGCR, which act as rate‐limiting enzymes for FA and sterol synthesis in a variety of eukaryotes.^140^ In addition, the nuclear receptor HNF4 alpha (HNF4α), a key regulator in mitochondrial FA β‐oxidation, cholesterol and bile acid metabolism, and lipoprotein metabolism,^141^ is also phosphorylated by AMPK. AMPK‐induced phosphorylation in the HNF4α protein reduces its ability to form homodimers, a process needed for binding to DNA, and thus enhancing the turnover of HNF4α.^142^ In astrocytic tumors, AMPK was shown to contribute to lipid metabolic reprogramming by reducing energy expenditure, through reduced *de novo* fatty synthesis and increased extracellular lipid internalization.^143^ SREBP1 is an important transcriptional regulator of lipid metabolism. AMPK can phosphorylate a conserved serine near the cleavage site within the SREBP1 protein that suppresses its activation leading to reduced lipogenesis.^144^ It was reported that Sirtuin1 (SIRT1), an NAD+‐dependent histone protein deacetylase that plays a vital role in lipid metabolism regulation, acts in concert with AMPK to regulate lipid metabolism.^145^ AMPK is an important regulator of lipid metabolism that appears to act differently based on the tissue context.

#### 3.2.7 The Notch signaling pathway

The Notch signaling pathway regulates a variety of key cellular processes, such as cell‐fate determination, proliferation, differentiation, and intercellular communication.^146^ A recent study identified a role for Notch in regulating lipid metabolism that was linked to tumor initiation. Notch‐mediated lipid metabolic reprogramming plays a central role in the initiation of liposarcomas. Adipocytes showing activation of the Notch pathway undergo a dedifferentiation with associated lipid metabolic dysfunction that eventually develops into liposarcoma. Inhibition of Notch signaling can lead to reduced lipid uptake and FA oxidation. A potential mechanism was suggested that involves a Notch‐driven inhibition in the production of PPARγ ligands that act as critical regulators of lipid homeostasis in adipocytes. Synthetic supplement of PPARγ was shown to partially reverse Notch activation‐induced transformation of adipocytes.^147^ In endothelial cells, Notch signaling pathway was reported to regulate the expression of endothelial lipase, an enzyme linked to FA transport across the vessel wall.^148^ The general role of Notch signaling in cancer is largely unexplored, and further study is required to determine whether other molecular mechanisms may contribute to Notch‐driven lipid metabolic reprogramming in cancer.

#### 3.2.8 The STAT3 signaling pathway

STAT3 belongs to the family of STAT transcriptional factors that participate in the regulation of a variety of cellular process, including proliferation, differentiation, inflammation, and stemness.^149^ Studies have revealed a regulatory role for STAT3 in lipid metabolism in lipocytes.^150^ Recent reports have shown that STAT3‐mediated lipid metabolism also plays essential role in cancer progression. STAT3 was found to inhibit cell ferroptosis via suppression of expression of acyl‐CoA synthetase long‐chain family member 4 (ACSL4), an enzyme that contributes to ferroptosis by enriching membranes with long polyunsaturated FAs.^151^ It was further shown that STAT3 can cooperate with androgen receptor (AR) to induce expression of cell cycle‐related kinase (CCRK), and thus, enhance the tumorigenicity of liver cancer. Mechanistically, CCRK was shown to increase levels of the mature form of SREBP1 in the nucleus via GSK3β/mTORC1 signaling resulting in increased lipid *de novo* synthesis and uptake.^152^ In ovarian cancer, STAT3 was found to promote FA uptake, driving activation of FABP4 that helped fuel cancer cell growth.^153^ In breast cancer, JAK/STAT3 was found to promote FAO in cancer stem cells via directly binding to the promoter of carnitine palmitoyltransferase 1B (CPT1B) that results in transcriptional activation of a key enzyme needed for FAO. Decreased levels of FAO were observed after STAT3 inhibition, while supplementation with the medium‐chain FA (myristic acid), which can bypass the need for CPT1B, reversed the inhibition of tumor spheroid formation and drug resistance induced by JAK/STAT3 inhibition. These findings suggest that STAT3‐mediated lipid metabolic reprogramming is essential for maintaining stemness properties and drug resistance.^154^

### 3.3 Noncoding RNA‐mediated lipid metabolic reprogramming in cancer

Noncoding RNAs (ncRNAs), mainly long noncoding RNAs (lncRNAs) and microRNAs (miRNAs), have been defined as important regulators of lipid metabolism in different types of cancers. A lncRNA HULC, highly upregulated in liver cancer, was shown to promote abnormal lipid metabolism through suppression of miR‐9, which normally drives the methylation of CpG islands in the PPARA promoter.^155^ In primary cervical cancer, the lncRNA LNMICC was found to promote LN metastasis by reprograming FA metabolism by recruiting NPM1 to the promoter of the FA binding protein FABP5.^156^ LncRNA SPRY4‐IT1 is aberrantly overexpressed in melanoma and was shown to control the expression of lipin2 and O‐acyltransferase 2 (DGAT2), and increase levels of acyl carnitine, fatty acyl chains, and triacylglycerol (TAG).^157^ Nuclear‐enriched abundant transcript 1 can bind to miR‐124‐3p and thereby disrupt lipolysis in HCC cells by modulating adipose triglyceride lipase (ATGL) and enhance HCC proliferation.^158^ The lncRNA MACC1‐AS1 has been reported to facilitate metabolic plasticity, and was further revealed to promote FAO‐dependent stemness and chemoresistance by antagonizing miR‐145‐5p in gastric cancer.^159^ Zhang and colleagues have reported that lncRNA LINC01138 is highly expressed in ccRCC. Through interaction with protein arginine methyltransferase 5 (PRMT5), LINC01138 was shown to increase arginine methylation and to stabilize SREBP1 leading to lipid desaturation and proliferation of ccRCC.^160^ In lung cancer, lncRNA HAGLR was shown to regulate FA synthase and to modulate MMP 9 and P‐21 expression.^161^

In addition, miR‐195 can directly target acetyl‐CoA carboxylase alpha (ACACA), FASN, HMGCR, and CYP family 27 subfamily B member 1 (CYP27B1) resulting in modulation of *de novo* GA/cholesterol synthesis and the inhibition of proliferation and invasion of breast cancer cells.^162^ miR‐185 and 342 can inhibit SREBP‐1 and 2 expression and downregulate FASN and HMGCR that will, in turn, influence lipogenesis and cholesterogenesis in prostate cancer.^163^ Glucose‐6‐phosphate‐dehydrogenase (G6PD), the rate‐limiting enzyme in the pentose phosphate pathway (PPP), was found to be increased often in human malignancies leading to the generation of precursors for nucleotide and lipid synthesis. miR‐122 has been shown to dysregulate G6PD in HCC by directly interacting with its 3′UTR and to further inhibit PPP.^164^ miR‐449 was also shown to control lipogenesis and cholesterogenesis in HCC. miR‐449 was reported to inhibit SIRT1 and SREBP‐1c and to further downregulate expression of target genes FASN and HMGCR.^165^ ATP citrate lyase (ACLY) is an enzyme that initiates *de novo* lipid synthesis. In osteosarcoma, prostate, cervical, and lung cancers, miR‐22 was reported to directly downregulate ACLY through posttranscriptional effects and was associated with tumor growth and metastasis.^166^ In addition, miR‐182 can suppress pyruvate dehydrogenase (PDH) kinase 4 (PDK4) and promote lung tumorigenesis through increased ACLY and FASN levels.^167^ In gastric cancer, the expression of miR‐422a can help drive a metabolic shift from aerobic glycolysis to oxidative phosphorylation by decreasing pyruvate dehydrogenase kinase 2 (PDK2) and thereby modulate *de novo* lipogenesis.^168^ Cheng et al reported that miR‐148a deletion can enhance lipid metabolic disorder and accelerate DEN‐induced hepatocarcinogenesis through regulation of lipid metabolism genes, such as PGC1α, Sirt7, HMGCR in murine liver physiology, and hepatocarcinogenesis.^169^ These findings strongly suggest a central role for ncRNAs in modulating the lipid metabolism of cancer cells.

## 4 LIPID METABOLISM AND TUMOR MICROENVIRONMENT

The TME is composed of immune cells, fibroblasts, endothelial cells, lipocytes, and extracellular matrix. The constant cross talk between the normal cells of the TME with the cancer cells represents an important component of tumor growth and progression. Lipids appear to strongly contribute to this cross talk. The stromal cells comprising the TME can also undergo lipid metabolism rewiring that helps them adapt to the stressful environment. Thus, the lipid regulation found within the TME appears quite dynamic. This includes the tumor and stromal cells, as well as regulation of lipid uptake from peripheral circulation.^170^ The lipids within the TME can strongly regulate the proliferation of cancer cells and help shape the function of the cancer‐associated stromal cells, especially immune cells.

### 4.1 Microenvironment‐mediated lipid metabolic reprogramming in cancer cells

The work to date suggests that the TME helps drive the lipid metabolic reprogramming that occurs in cancer cells, and thus, can be seen as playing an essential role in the adaption of the cancerous cells to the TME. The anoxic and acidic microenvironment within solid tumors is associated with tumor aggressiveness and treatment resistance. Increased uptake and accumulation of lipoprotein is observed in cancer cells exposed to hypoxia and acidosis, and this lipid enrichment phenotype is associated with an increased ability to form spheroids *in vitro* (a measure of stemness characteristics) and the ability to undergo metastasis.^171^ Cancer cells adapted to acidic pH show increased generation of FAO‐derived acetyl‐CoA in their mitochondrial that helps fuel the tricarboxylic acid (TCA) cycle and lead to an enhanced mitochondrial protein acetylation. This enhanced acetylation of mitochondrial protein can inhibit the activity of pyruvate‐/malate‐driven complex I, and decrease generation of ROS eventually contributing to the proliferation of acidic pH‐adapted cells.^172^ Kondo et al reported that acid stimulation could enhance cholesterol biosynthesis in cancer cells via increasing activity of SREBP2, and thus facilitating their survival within the acidic TME.^173^

**FIGURE 3:**
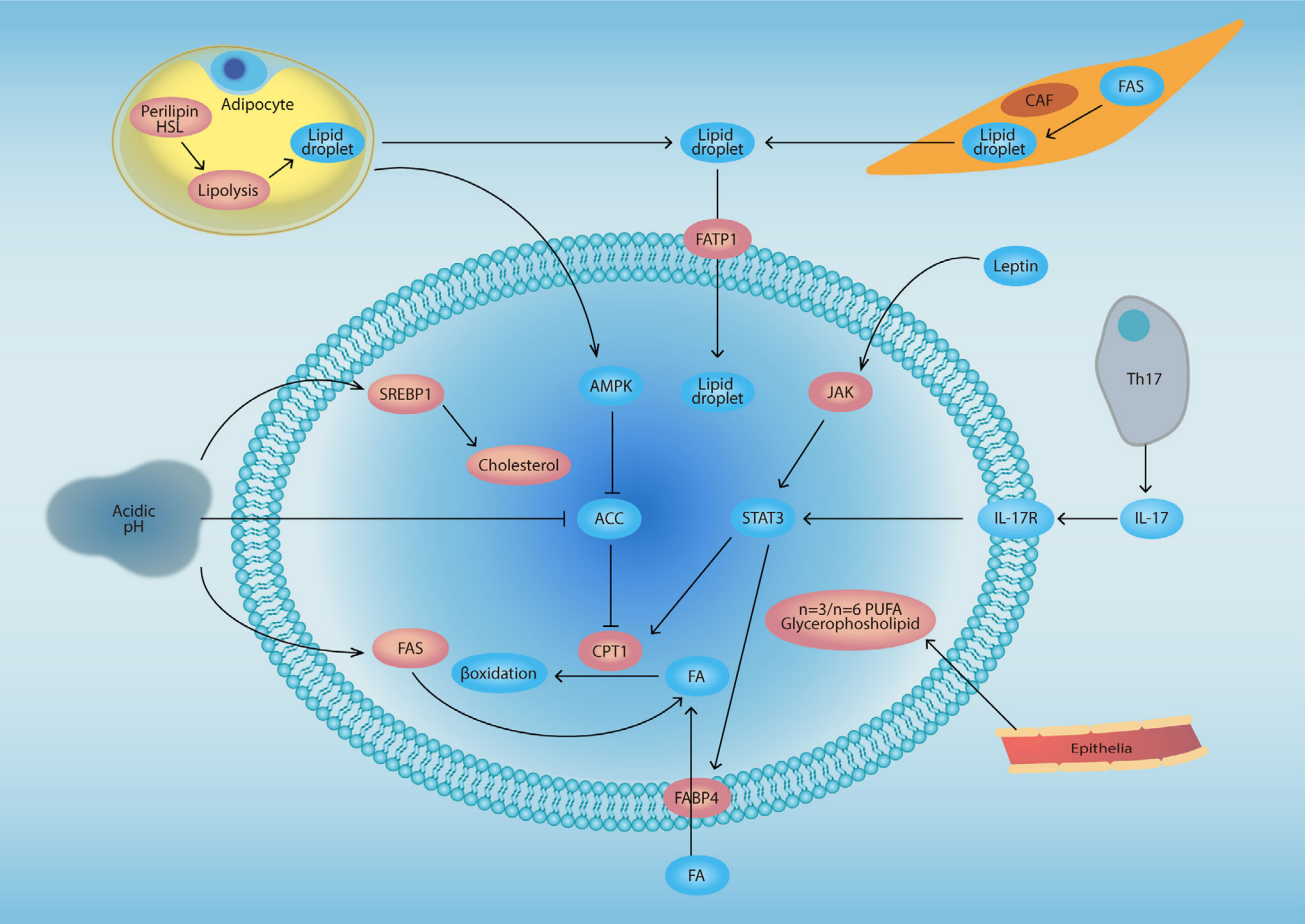
Microenvironment‐mediated lipid metabolic reprogramming in cancer. Acidic pH in the TME contributes to enhanced cholesterol and FA synthesis in cancer cells. Lipocytes can activate AMPK to facilitate FAO in tumor cells. In addition, enhanced lipolysis by lipocytes generates lipid droplets that can be absorbed by cancer cells. CAFs also show enhanced lipolysis that helps fuel cancer cells with lipid droplets. Leptin in the TME and TH‐17‐secreted IL‐17 can promote FA uptake and FAO via activation of STAT3 signaling. Epithelial cells can increase the level of PUFA and glycerophosholipid in cancer cells Abbreviations: AMPK, adenosine 5‘‐monophosphate (AMP)‐activated protein kinase; ACC, acetyl‐CoA carboxylase; CAF, cancer‐associated fibroblast; FATP1, fatty acid transport protein 1; STAT3, signal transducer and activator of transcription 3.

Noncancer stromal cells in TME can promote tumor growth and metastasis through the paracrine secretion of factors, such as cytokines, growth factors, Wnt ligands, and MMPs,^18^ which help trigger lipid metabolic reprogramming in the cancer cells. In addition, the stromal cells share soluble metabolites with the cancer cells that can help facilitate tumor progression. It was reported that interaction between endothelial cells and cancer cells can lead to increased levels of cytosolic n‐3 and n‐6 polyunsaturated FAs (PUFAs) and glycerophospholipids—lipids linked to cancer progression.^174^ A series of studies have demonstrated that adipocyte‐derived FAs play an essential role within the cancer cell niche. Lipid‐mediated cross talk between adipocytes and cancer cells contribute to cancer progression, including fostering the stemness properties linked to cancer progression, and helping maintain distant metastasis. Yang et al reported that adipocyte‐derived lipids can be transferred to breast cancer cells and the interaction of tumor cells with adipocytes can also lead to an increased expression of the key metabolic enzyme lipase ATGL and intracellular FA trafficking protein FABP5, which are thought to cooperate to promote breast cancer progression.^175^ Adipocytes can increase lipid droplet accumulation in cancer cells and thus promote cancer cell growth. Adipocytes enhance their production and release of free FAs in response to increased expression and activity of lipolysis‐associated genes, such as perilipin and hormone‐sensitive lipase (HSL). The interaction between adipocytes and ovarian cancer cells can promote ovarian cancer cell metastases and proliferation within the omentum, driven through adipocyte activation of FA β‐oxidation in ovarian cancer cells. Wang et al reported that adipocytes can potentially contribute to the stemness properties of breast cancer cells by secretion of leptin in breast cancer leading to increased expression of CPT1B gene expression and activation of FAO in the cancer cells. FAO inhibition has been shown to reduce tumor‐spheroid formation in breast cancer stem cells, while activation of FAO restores tumor‐sphere formation ability in STAT3‐inhibited cells.^176^ These studies strongly suggested that TME plays an essential role in the lipid metabolic reprogramming of cancer cells and helps facilitate tumor progression (Figure 3).

### 4.2 Lipid metabolism in cancer‐related stromal cells

Tumor stromal cells, which comprise ∼50% of the cell population in tumor tissues, can also undergo lipid metabolic reprogramming. CAFs are important stromal cells that promote cancer growth and progression. These cells can help modulate lipid metabolism, for example, through PGE production. In neuroblastoma, CAFs were found to express increased PGE that, in turn, was linked to enhanced tumor growth, immune suppression, and increased angiogenesis.^177^ The EMT‐related factor Snail may partially account for the enhanced PGE secretion by CAFs in response to TGF‐β stimulation.^178^

Tumor infiltrating lymphocytes (TILs) can recognize and kill malignant tumor cells and represent an important endogenous check on tumor growth. These important effector cells often lose their ability to kill tumor cells due to exhaustion, a phenomenon that remains poorly understood. Lipid metabolism reprogramming in TIL can be triggered by the TME, and lipids and their derivatives can impact TIL function. In the nutrient competitive TME, TILs must rewire their metabolism to maintain their functional activity. Lipid metabolic patterns change during the functional maturation and activation of T cells. T cells are represented by a variety of linages of effector cells: Th1, Th2, Th17, Treg, cytotoxic T lymphocytes (CTLs), and diverse memory cells. Normally, the generation of T effector cells is accompanied by decreased FA oxidation, enhanced glycolysis, and FA synthesis. Treg cells and CD8+ T memory cells rely on FAO for their generation.^179^, ^180^, ^181^ mTOR and AMPK help shape the metabolic pathways in T cells. FA synthesis in TILs is driven through the PI3K/AKT/mTORC1/SREBP axis, while AMPK can suppress mTORC1 through phosphorylation of raptor, and by this, promote generation of the TSC1‐TSC2 complex and an increase in FAO.^182^

In the TME, Treg cells transport intracellular FA through FABP5 to fuel FAO. The inhibition of FA metabolism by blocking FABP5 can damage mitochondrial integrity and suppress the expansion and proliferation of Treg cells.^183^ Unlike Treg cells developed *in vitro*, tumor‐bed‐derived Tregs mediate FA synthesis through mTOR/SREBP signaling that accumulates intracellular lipids and promotes proliferation.^184^ Normally, CTL and other effector T cells show pronounced mTOR signaling and feature robust glycolysis and lipid synthesis. In the TME, T effector cells preserve their FAS activity and FAS plays an important role in maintaining their function. Reduction of FA synthesis by either raptor deficiency or inhibition of SREBP will reduce CD8+ T cell growth and proliferation i*n vitro*.^180^, ^182^, ^185^ Deficiency of ACC1, a key enzyme in lipid synthesis, will decrease antigen‐specific expansion and accumulation of CD8+ T cells in mice infected with monocytogenes.^185^ However, within the metabolically competitive environment that characterizes the TME, glycolysis by effector T cells is suppressed as due to a shortage of glucose, which can lead to their exhaustion.^186^ These cells may reboot to FAO as a mean of maintaining function in response to lipid/PPAR signaling. PPAR signaling plays an important role in maintaining CD8+ T cell antitumor function by reactivating FAO. Free FA activates PPAR signals and, in turn, leads to the upregulation of CPT1, and finally, an increase in the FAO of CD8+ T cells. A recent study described increased FA uptake and FAO resulting from enhanced activation of PPARα signaling in glucose starved and oxygen deprived CD8+ T cells. Thus, promoting FAO in CD8+ T cells through application of a PPARα agonist may enhance antitumor effects and facilitate anti‐PD1 therapy.^187^ More extensive studies with PPARα agonists will help determine the potential of using this biology as a means of T cell reinvigoration.^188^ Lipid metabolic reprogramming is also linked to PD1‐induced T cell exhaustion. T cells receiving PD1 signals undergo metabolic reprogramming characterized by arrogated metabolism of glucose and amino acids, and increased FAO used for energy generation.^189^ The degree of exhaustion and the ability of these cells to reinvigorate may depend on the supply of lipids, the only source of energy generation by FAO. The general metabolism of TILs can be influenced by cholesterol. Previous studies have shown that T cell receptor (TCR) activation promotes activation of the cholesterol biosynthesis pathway and increased cholesterol levels in T cells, which is driven through the SREBP2 pathway. Preventing cholesterol esterification by inhibition of ACTA1 can help drive the biosynthesis of cholesterol in CD8+ T cells and enhance their antitumor effector function against myeloma.^190^

Myeloid cells, including MDSCs, tumor‐associated macrophages (TAMs), and dendritic cells (DCs), represent an important component of the TME. These cells play a role in the inhibition of antitumor T cell immunity and may undermine tumor immune therapy. DCs initiate and maintain TIL dependent antitumor immunity. An enrichment of cellular lipid is related to a compromised capacity of DCs to assist in the activation of TIL. DCs in the tumor milieu increase their cellular lipids by absorbing lipids from the environment and by increasing FAs synthesis. A result of increased FAs synthesis by activation of FASN, or increased FAs uptake by upregulating SRA, will lead to an inhibition in the ability of DCs to support antitumor T cells in cancer.^191^, ^192^ A disruption of DCs lipid hemostasis can be triggered by TME lipid metabolites. Lipid peroxidation products can trigger ER stress in ovarian‐cancer‐associated DCs. The ER stress‐related protein XBP1 can upregulate the expression of TG biosynthesis‐related target genes, and subsequently lead to an increase the cytosolic lipid content of DCs. In addition to lipid peroxidation products, oxysterol in the TME can activate LXR‐α in DCs. This can lead to a decrease in expression of CCR7 on the DCs surface and thereby a reduction in the ability of the mature DCs to migrate to LNs resulting in less antitumor immunity.

TAMs can also undergo lipid metabolism reprogramming during their activation and maturation. The classification of TAMs includes antitumor M1‐like TAMs and protumor M2‐like TAMs. Generally, LPS and IFN‐γ activated, M1‐like TAMs increase FAs synthesis through citrate accumulation by transcriptional repression of IDH, while IL‐4/IL‐13 activated, M2‐like TAMs increase FAO driven by STAT6/PGC1β.^193^ The TME shapes TAM metabolism and function through the presence of metabolites and cell components. Treg cells can help enhance SREBP‐1 expression in M2‐like TAMs through repression of their interferon‐γ secretion by CD8+ T cells. This can lead to an activation of FA synthesis and polarization of the TAMs to a M2‐like TAM phenotype.^194^ The accumulation of intracellular lipids is important for M1‐like TAMs. Previous studies have shown that intracellular FAs enhance the cytotoxic activity of M1‐like TAMs. Increased cytotoxic activity was shown in murine peritoneal macrophages that were enriched with intracellular lipids. The upregulation of the FA binding protein EFABP in M1‐like TAMs can improve their IFN‐β‐induced antitumor activity and increase the lipid droplet content of the TAMs. Stimulating M1‐like TAMs with an EFABP activator also elevates lipid droplet formation and IFN‐β secretion.^195^, ^196^ However, extracellular lipids and lipid metabolites can redirect the polarization of TAMs. Recent studies have shown that extracellular FAs, especially unsaturated FAs such as oleate, are able to polarize bone‐marrow myeloid cells into M2‐like TAMs, as well as increasing lipid‐derived PGE through upregulation of COX‐2,^197^ which can contribute to the immune suppressive environment of the TME. Another study has shown TAM transport of extracellular lipid into the cytosol through CD36 to fuel FAO. High levels of FAO can drive polarization toward M2‐like TAMs through phosphorylation of JAK1 and STAT6 activation. Inhibition of FAO by genetically ablating CD36 was shown to suppress tumor‐promoting TAM activity.^198^ TAMs can alter their cholesterol metabolism in response to the TME. Ovarian cancer cells can stimulate the membrane‐cholesterol efflux of TAMs through expression of the ABC transporters. Cholesterol efflux can promote the IL‐4‐mediated reprogramming of TAMs and inhibit IFN‐γ‐related gene expression.^199^ On the one hand, unsaturated FAs appear to be able to reprogram TAMs through IL‐4 mediated pathways and promote FAO that can suppress antitumor activity, and help drive macrophage polarization toward M2‐like TAMs. On the other hand, increasing lipid metabolism mediated by EFABP is related to an accumulation of intracellular FA, and IFN‐γ mediated antitumor activities of M1‐like TAMs. Surprisingly, activation of FAS via SREBP was recently described in M2‐like TAMs, with assistance by Treg cells that suggests cell‐cell interactions may be important in some metabolism reprogramming. Further work will help elucidate the impact of TME on lipid metabolism and immune regulation of TAMs. Taken together, the TME has a complicated influence on TAM metabolic reprogramming. In the TME, MDSCs increase FA uptake and use FAO as a primary source of ATP production.^200^ Interference with FAO can block the suppressive activity of MDSCs. MDSCs increase their lipid uptake through an upregulation of lipid transport receptors that is mediated through stimulation by tumor‐derived G‐CSF and GM‐CSF, and subsequent signaling through STAT3 and STAT5. Inhibition of lipid uptake by genetic depletion of the FA translocase CD36, or inhibition of the STAT3 or STAT5 signal, results in an impaired immunosuppressive function in MDSCs and delayed tumor growth.^201^ In this context, in human and mouse PMN‐MDSCs, STAT5 activates FATP2 that, in turn, drives lipid uptake and PGE2 synthesis. Deletion of FATP2 abolishes the activity of PMN‐MDSCs.^28^ Lipid metabolites in the TME can also modulate MDSC accumulation and enhance their immunosuppressive activity. PGE2 has been reported to induce the differentiation of MDSCs and to enhance MDSC immune suppression through the EP2 receptor. Oxysterol can recruit neutrophils through the CXCR2 receptor.^202^ Other studies have reported that MDSCs can secrete arginase through PGE2/EP4 stimulation that can block T effector cell function in lung cancer.^203^

### 4.3 Lipid derivatives can regulate tumor immunogenicity

**FIGURE 4:**
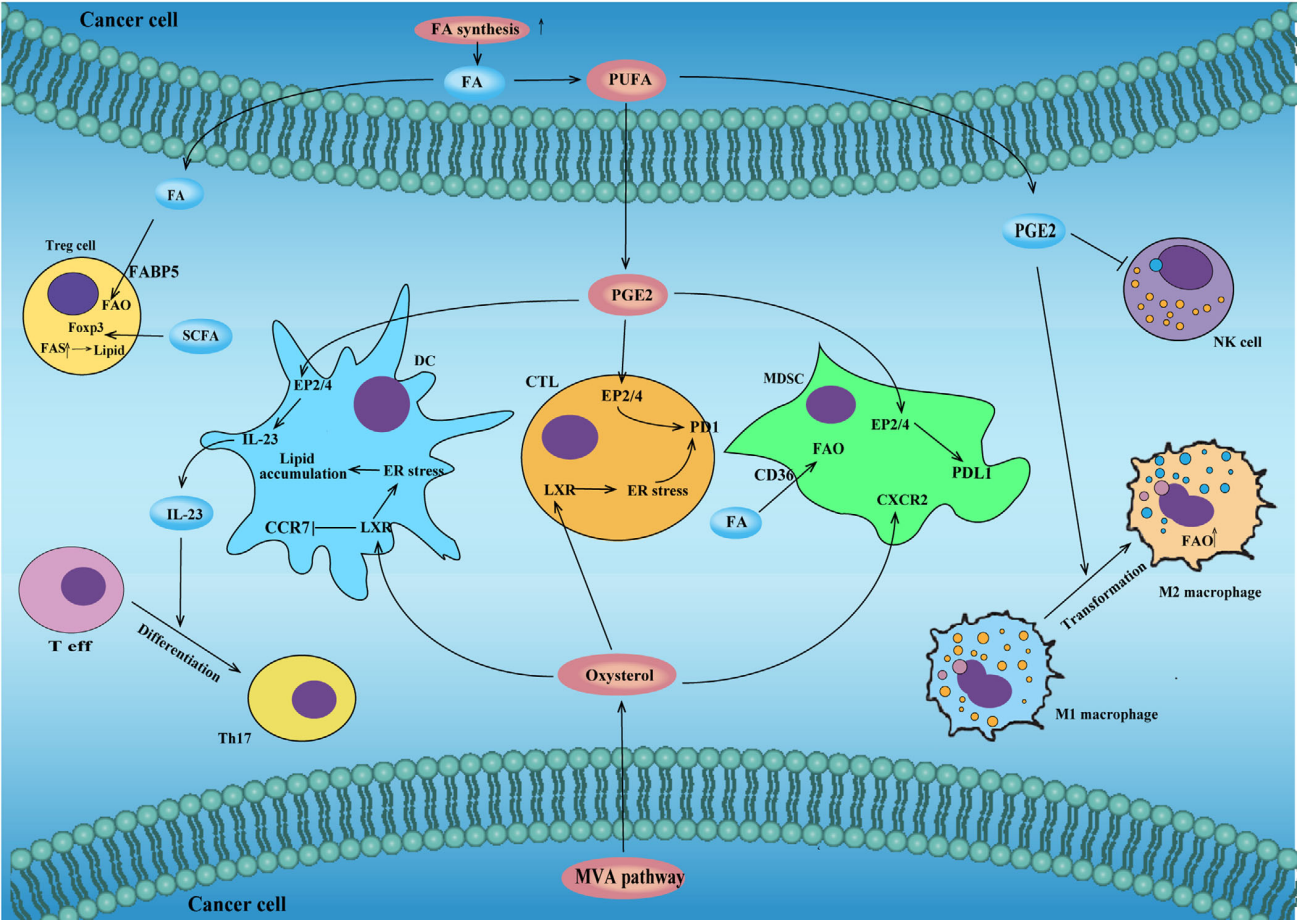
Lipid metabolism reprogramming influences tumor immunogenicity. Increased FA synthesis in cancer cells contributes to lipid storage in TME, which fuels FAO in Treg cells. SCFA can also promote the development of Treg cells via inducing Foxp3 expression. Cancer cell‐secreted PGE2 can suppress NK cell cytotoxicity and NK cell differentiation. PGE2 can also lead to increased expression of PD1 in CTL and increased expression of PD‐L1 in MDSC. PGE2 can inhibit NK cell function and promote the polarization of macrophages to M2 macrophages. PGE2‐secreted by mammary gland tumor cells can also the activate EP2 and EP4 receptors in DCs to stimulate the generation of IL‐23, a critical cytokine for Th17 cell survival and expansion. Oxysterol from cancer cells can inhibit T cell antitumor immunity via induction of immune check point expression and T cell exhaustion. Oxysterol may activate LXR to decrease the expression of CCR7 on the DC surface, preventing their migration to secondary lymphatics and thus reducing potential antigen presentation. Oxysterol can interact with CXCR2 to recruit MDSC into the TME Abbreviations: CCR7, C‐C motif chemokine receptor 7; CXCR2, C‐X‐C motif chemokine receptor 2; DC, dendritic cells; EP2/4, prostaglandin E receptor 2/4; ER, endoplasmic reticulum; FA, fatty acid; FABP5, fatty acid binding protein 5; FAO, fatty acid oxidation; FAS, fatty acid synthesis; Foxp3, forkhead box P3; LXR, liver X receptor; MDSC, myeloid‐derived suppressor cells; NK cell, natural killer cell; PD1, programmed cell death 1; PDL1, programmed cell death 1 ligand 1; PGE2, prostaglandin E2; PUFA, polyunsaturated fatty acid; Th17, T helper cell 17.

Immune escape is a hallmark of cancer. Great effort has been made to better understand the immune microenvironment of tumor cells. Increasing evidence suggested that lipid metabolism rewiring in tumor cells and TME exerts critical regulatory effects on tumor immunogenicity (Figure 4). Lipid‐derived signal molecules, termed lipid mediators, also play an important role in shaping the TME toward an inflammatory or immunosuppressive state. The concentration of S1P present in cancer tissue contributes to a link between cancer progression and chronic inflammation in colitis‐associated cancer, by inducing S1P/S1PR1/STAT3‐mediated production of IL‐6.^204^ In addition, the S1P‐S1PR1 axis also acts as a modulator of the tumor immune response by attracting monocytes and driving a M2 phenotype polarization in macrophages.^205^ PGE2 is a well‐characterized lipid mediator. It is an unsaturated FA generated from AA by the enzyme COX‐2. PGE2 is secreted by thyroid cancer cells that help them escape immune attack via suppression of natural killer (NK) cell cytotoxicity and NK cell differentiation.^206^ In gastric cancer, tumor‐derived PGE2 also promotes tumor progression by suppressing proliferation and increasing apoptosis of NK cells.^207^ In addition, PGE2 also contributes to PD‐L1 expression in TAMs and MDSCs, which facilitate immune suppression in tumor host.^19^ PGE2 was also reported to upregulate PD‐1 expression through EP2/4 receptors in CD8+ CTLs resulting in enhanced immune tolerance to lung cancer.^208^ PGE2 signaling can influence the effector function of TAMs that mediate a switch from M1‐like TAMs to M2‐like TAMs in cancer driven through EP4 receptor signaling. Treating peritoneal macrophages with an EP4 agonist will enhance M2 macrophage polarization, while EP4‐deficient macrophages showed less sensitivity to polarization.^209^ Moreover, in hypoxic conditions, PGE2 may be a potential inducer of VEGF‐C/D secretion in M2 TAMs activated through EP2, which leads to lymphatic endothelial cell sprouting.^210^ PGE2‐secreted by mammary gland tumor cells has been shown to activate the EP2 and EP4 receptors in DCs, leading to the generation of IL‐23, a critical cytokine for Th17 cell survival and expansion.^211^ The contribution of PGE2 to tumor immunogenicity may depend to a degree on the tissue context. Cholesterol metabolism also plays a significant role in regulating antitumor immunity. Yang et al reported that pharmacologically inhibiting the cholesterol esterification enzyme ACAT1 contributes to an enhanced proliferation and effector cytotoxicity of CD8+ T cells. It is thought that ACAT1 inhibition leads to an increase in cholesterol in the plasma membrane of CD8+ T cells, and enhanced T‐cell receptor clustering and signaling.^190^

Recently, a regulatory role of cancer lipid metabolism in rewiring general tumor immunogenicity has been observed. In melanoma cells, elevated levels in the lipid oxidative metabolic state were shown to increase the sensitivity of the melanoma cells to T‐cell‐mediated killing via increased expression of the major histocompatibility complex (MHC) Class I molecules that present antigen to cytotoxic T cells.^212^ Beyond effects on antigen presentation, lipid metabolites in cancer can also directly reprogram immune cells within the TME. For example, cancer cells can secret cholesterol metabolites that regulate immune cell function. The effect of cholesterol on immune cells is complicated, with intrinsic or extrinsic cholesterol shown to have different effects on TILs. SREBP‐mediated upregulation of intrinsic cholesterol biosynthesis is essential for CD8+ T cell proliferation and effector function,^180^ whereas cholesterol or its oxidized products accumulated in the TME may inhibit T cell antitumor immunity. Cholesterol accumulation in the TME may increase immune checkpoint molecule expression and foster T cell exhaustion via increased ER stress sensor‐XBP1‐caused upregulation of PD1 and 2B4.^213^ LXR, the nuclear receptor for oxysterols (cholesterol‐oxidized products), can negatively regulate T cell function, so oxysterols in the TME may lead to activation of LXR and thus inhibition of antitumor immunity by T cell.^214^ Oxysterols may induce LXR sumoylation and thereby inhibit the IL‐9 expression essential for CD8+ T cell polarization into the IL‐9‐secreting version of these effector cells with better antitumor effector function.^215^ In pancreatic neuroendocrine tumors, HIF1α can activate transcription of the enzyme Cyp46a1 that helps generate the oxysterol 24‐hydroxycholesterol (24S‐HC) that can further recruit the neutrophils that foster neoangiogenesis. The inhibition of oxysterols can reduce tumorigenesis by dampening the 24S‐HC–neutrophil axis.^216^ In breast cancer, 27‐hydroxycholesterol accounts for the high‐cholesterol‐diet‐induced tumor metastasis that has been described. Mechanistically, the enzyme CYP27A1‐driven generation of 27‐hydroxycholesterol at distal metastatic sites helps attract PMN‐neutrophils and γδ‐T cells, and at the same time decrease the recruitment of cytotoxic CD8^+^ T lymphocytes, thus favoring the formation of a premetastatic niche.^217^

Thus, it can be seen that lipid metabolism participates in tumor immune escape via regulation of MHC‐mediated antigen presentation, activation of immune suppressive cells, and the dysfunction of cytotoxic effector T cells.

## 5 THERAPEUTIC STRATEGIES TARGETING LIPID METABOLISM REPROGRAMMING IN CANCER

Lipid metabolic rewiring is essential for tumor survival and proliferation, and therapeutic intervention targeting lipid metabolic reprogramming in cancer may represent an effective means of controlling tumor growth. Lipid metabolic pathways, lipid metabolism‐related enzymes, and molecular mechanisms underlying lipid metabolic rewiring all represent potential therapeutic targets for anticancer strategies.

### 5.1 Targeting FA metabolism

Based on the essential role of FAs in tumor cell survival and growth, limiting the availability of FAs may represent a therapeutic strategy.^4^ For example, the breakdown of TGs stored as LDs in cancer cells represents an important intracellular source of FAs. The small molecule JZL184 inhibits MAGL, a rate‐limiting enzyme in TGs decomposition, leading to lower cytoplasmic FAs levels and reduced pathogenicity of prostate cancer cells.^218^ Therapies targeting FAs in cancer can be divided into three general strategies: (a) blocking de novo FAs synthesis, (b) blocking FAs uptake, and (c) blocking FA utilization. Anticancer drugs that target FA metabolism are detailed in Table 1.

**TABLE 1:** Summary of anticancer drugs targeting fatty acid metabolism

Target	Compound	Type of cancer	Preclinical model or clinical trial	Refs
FASN	C75	Breast cancer, GBM, RCC, mesothelioma, glioma, lung cancer, and melanomas	Xenografts	^221^, ^278^, ^279^
	Cerulenin	Ovarian cancer and breast cancer	Xenografts	^280^
	Orlistat	Prostate cancer, melanoma, glioma endometrial cancer, melanomas, and OTSCC	Xenografts	^281^, ^282^, ^283^, ^284^, ^285^, ^286^
	Triclosan	Prostate cancer and breast cancer	Xenografts	^287^
	amentoflavone	Breast cancer	Xenografts	^288^
	EGCG	Lung cancer, breast cancer	Xenografts	^279^, ^289^
	TVB‐3166	Ovarian cancer	Xenografts	^290^
ACLY	SB‐204990	Lung cancer	Xenografts	^227^, ^291^
	LY294002	Lung cancer	Xenografts	^292^
ACC	TOFA	HNSCC, ovarian cancer, colon cancer, and RCC	Xenografts	^293^, ^294^, ^295^, ^296^
	Metformin	Lung cancer, prostate cancer, and ovarian cancer	Xenografts	^297^, ^298^
	AICAR	HCC, prostate cancer, and cervix cancer	Xenografts	^299^
	ND‐654	HCC	Xenografts	^223^
	ND‐646	NSCLC	Xenografts	^300^
SCD	BZ36	Prostate cancer	Xenografts	^240^
	A939572	Renal cancer	Xenografts	^24^, ^301^, ^302^
	CAY‐10566	HCC, breast cancer, HNSCC, and ovarian cancer	Xenografts	^118^, ^303^, ^304^, ^305^
	CVT‐11127	HCC	Xenografts	^306^
	MF‐438	Lung cancer	Xenografts	^307^, ^308^
	T‐3764518	CRC and mesothelioma	Xenografts	^308^, ^309^
CPT1	Etomoxir	Prostate cancer, breast cancer, and glioblastoma	Xenografts	^110^, ^237^, ^238^, ^310^
	Ranolazine	Prostate cancer	Xenografts	^311^
	Perhexiline	Breast cancer, prostate cancer, and lymphocytic leukemia	Xenografts	^237^, ^312^, ^313^
ACSS	Triacscin C	Lung cancer, colon cancer, and brain cancer	Xenografts	^314^

Abbreviations: ACC, acetyl‐CoA carboxylase; ACLY, ATP citrate lyase; ACSS, acetyl‐CoA synthetase; EGCG , (‐)‐Epigallocatechin 3‐gallate; FASN, fatty‐acid synthase; GBM, glioblastoma multiform; HCC, hepatocellular carcinoma; HNSCC, head and neck squamous cell carcinoma; NSCLC, nonsmall‐cell lung cancer; OTSCC, oral tongue squamous cell carcinom; RCC, renal cell cancer; CRC, colorectal cancer; SCD, stearoyl‐CoA desaturases; CPT1, carnitine palmitoyltransferase 1

#### 5.1.1 Blocking *de novo* FA synthesis

Cancer cells typically show increased *de novo* synthesis of FA.^219^ Rate‐limiting enzymes for FAs synthesis, such as FASN, ACLY, and ACC, are significantly upregulated in cancer cells,^4^ and inhibition of these enzymes can decrease the proliferation and growth of cancer cells.^4^, ^9^, ^219^ Preclinical studies have identified an antitumor effect of compounds that target FA metabolic pathways. Cerulenin, a natural metabolite of cephalosporin cyanobacterium, is an FASN inhibitor.^220^ It blocks the synthesis of FAs in cells, and has cytotoxic effects on a variety of cancer cell lines. C75, an FASN inhibitor has more stable chemical properties than cerulenin, and also shows a strong inhibitory effect on a variety of human cancer cell lines, including breast cancer, prostate cancer, mesothelioma, and ovarian cancer.^221^ FA metabolic reprogramming contributes to cancer adaption to antiangiogenic treatments, and the use of FASN inhibitors can inhibit tumor regrowth and metastasis after sunitinib treatment withdrawal.^33^ Furutal et al reported that the combination of an FASN inhibitor with cyclophosphamide partially overcame the drug resistance caused by tumor hypoxia, suggesting that FASN inhibition may improve the efficiency of chemotherapy.^222^ Jiang et al reported that tumor‐cell‐intrinsic FASN can lead to an absence of T‐cell infiltrates and DC malfunction, and is associated with an immunosuppressive microenvironment in ovarian cancer. Thus, targeting FASN in combination with immunotherapy may show clinical benefit.^191^ ACC is a critical enzyme in FAs biosynthesis that is upregulated in cancer cells. Inhibiting ACC has an antitumor effect. In liver cancer, ND‐654, an ACC inhibitor, has been shown to suppress the development of cancer.^223^ Similar results have been observed in EGFRvIII human GBM cells. ACLY, a key cytoplasmic enzyme catalyzing citric acid to acetyl‐CoA, is overexpressed in GBM, colorectal cancer, breast cancer, nonsmall cell lung cancer, and HCC.^224^, ^225^, ^226^ The ACLY inhibitor SB‐204990 has been shown to inhibit the proliferation of lung cancer cells *in vivo* and *in vitro*.^227^ Some of the ACLY inhibitors have been demonstrated to block the synthesis of FAs,^228^ including citric acid analogues, such as difluorocitric acid and hydroxycitrate.^229^ Although some ACLY inhibitors have been validated, most of the studies to date are preclinical.

FASN, ACC, and ACLY are downstream target genes of the SREBPs transcription factors that are upregulated in cancer cells. The SREBPs may thus represent therapeutic targets. Previous studies have suggested that SREBP1 inhibition can reduce the proliferation and migration of a variety of tumor cells *in vitro* and *in vivo*. Fatostatin, a nonsteroidal diarylthiazole derivative, has been shown to inhibit the activation of SREBP1 by blocking the transport of SCAP from the ER into the golgi apparatus.^230^ In p53‐mutated prostate cancer, fatostatin was shown to inhibit cell growth, induce caspase‐dependent apoptosis, and inhibit G2/M cell cycle arrest.^231^ Clinical trials evaluating the potential antitumor efficiency of compounds inhibiting FA *de novo* synthesis are currently underway. The ACC inhibitor ND‐630 and the FASN inhibitor TVB‐2640 are undergoing phase I and II clinical trials (NCT03808558 and NCT02876796). In addition, a clinical study investigating the efficacy and safety of TVB‐ 2640 in combination with bevacizumab in patients with first relapse of high‐grade astrocytoma is underway (NCT03032484). Inhibiting *de novo* synthesis of FAs via targeting transcriptional regulators or the rate‐limiting enzymes for FA synthesis may represent targets for the inhibition of tumor growth.

#### 5.1.2 Blocking FA uptake

An increasing number of reports have shown that tumors enhance FA uptake from their environment, suggesting that blocking the FA uptake pathway may show efficacy for cancer treatment.^232^ Inhibition of CD36, a transmembrane protein that mediates FA uptake, has been shown to suppress tumor growth.^26^ Other proteins that contribute to an increased uptake of FAs in cancers, including LDLR, FATPs, and FABPs, are also upregulated in tumors.^233^ Melatonin can indirectly inhibit the function of FATPs to suppress tumor growth.^234^ FATP2 is also crucial for PMN‐MDSCs to acquire an immunosuppressive phenotype, and the inhibition of FATP2 can abrogate the suppressive activity of PMN‐MDSCs, thus improving the anticancer immunogenicity.^28^ Rao et al reported that the compound 5‐(benzylamino)‐2‐(3‐methylphenyl)‐1,3‐oxazole‐4‐carbonitrile can serve as an inhibitor of FABPs and inhibit mammary tumor growth.^195^ Studies have shown that the TME can serve as an important supplier of FAs for cancer cells, in addition it is also associated with increased lipolysis of cancer stromal cells, such as CAFs and adipocytes.^175^, ^235^ Understanding potential molecular mechanisms contributing to enhanced FAs in TME may pave the way to novel anticancer therapeutics.

#### 5.1.3 Blocking FA utilization

FAs perform diverse functions in cells, and as discussed, one of these is to supply energy through FAO. The overexpression of FAO‐related enzymes has been found in multiple types of malignancies, and their critical role in tumor progression has been demonstrated.^236^ CPT1 is the rate‐limiting enzyme of FAO, and is required for the rapid proliferation of cancer cells.^237^ CPT1 inhibitors, such as etomoxir and ranolazine, show antitumor efficacy in prostate cancer.^238^ A similar result has been observed in breast cancer^110^ and GBM.^237^ Etomoxir can improve the efficiency of chemotherapy in lymphoma when used in combination therapy.^238^ In addition, combined therapy targeting of the FAO and VEGF pathways was found to produce superior anticancer effects when compared with monotherapies, suggesting the efficacy of CPT1 inhibitors to overcome the antiangiogenic drug (AAD) resistance.^239^ These findings strongly suggest the potential of CPT1 as a therapeutic target for cancer treatment. Interestingly, FAO is essential for MDSCs function, so CPT1 inhibitor treatment may also inhibit the function of MDSCs, further enhancing its antitumor efficiency.^200^ Apart from FAO, FAs can also undergo desaturation to form MUFA, and this process is also critical for cancer cell survival. SCD1 is overexpressed in a variety of malignant tumors and is a critical enzyme that helps cancer cells transform saturated lipid into desaturated lipid, thus protecting the cell from saturated lipid toxicity‐induced cell death.^200^ Treatment with SCD1 inhibitors was found to disrupt the balance between MUFA and SFA and reduce the survival rate of tumor cells in xenograft models of prostate and lung cancer.^240^ To date, several compounds targeting SCD1 in tumor treatment, including CAY‐10566 and MF‐438, have been limited to preclinical trials, mainly due to the weight loss and toxic side effects on skin and eyes.^241^ Overcoming these toxic side effects represents a key point in the development of the next generation of SCD1 inhibitors.

### 5.2 Targeting cholesterol metabolism

Numerous studies have identified a critical role for cholesterol metabolism in cancer survival. Cholesterol metabolism intermediates and cholesterol metabolic reprogramming‐related genes, which are dysregulated and contribute to cancer progression, may represent promising therapeutic targets for cancer treatment. Anticancer drugs that target cholesterol metabolism are detailed in Table 2.

**TABLE 2:** Summary of anticancer drugs targeting cholesterol metabolism

Target	Compound	Type of cancer	Preclinical model or clinical trial	Refs
SREBPs	Fatostatin	Prostate cancer	Xenografts	^8^, ^231^
	25‐HC	Glioblastoma	Xenografts	^122^
	Betulin	Lung cancer	Xenografts	^315^
HMGCR	Statins	CRC, prostate cancer, and multiple myeloma	A cohort study	^316^, ^317^, ^318^
	Lipophilic statins	Melanoma	Xenografts	^319^
ACAT1	Avasimibe	Prostate cancer	Xenografts	^320^
	Avasimin	Prostate cancer and CRC	Xenografts	^321^
	Bitter‐melon extract	Breast cancer	Xenografts	^322^
LXR	RGX‐104	Melanoma and lung cancer	Xenografts	^323^
	LXR623	RCC	Xenografts	^324^
	SR9243	CRC and prostate and lung cancer	Xenografts	^325^
OSC	R048‐8071	CRC and pancreatic adenocarcinoma	Xenografts	^326^
Squalene synthase	Zaragonic acids	Lymphoma and lung cancer	Xenografts	^327^
CYP27A1	GW273297X	Breast cancer	Xenografts	^217^

Abbreviations: ACAT1, cholesterol acyltransferase 1; CYP27A1: cytochrome p450 27A1; HMGCR, HMG‐CoA reductase; LXR, liver X receptor; OSC, oxidosqualene cyclase; SREBPs, sterol regulatory element‐binding proteins; CRC, colorectal cancer; RCC renal cell carcanima

#### 5.2.1 Blocking cholesterol biosynthesis

Reducing the generation of cholesterol could reduce lipid raft formation, block energy storage, and reduce the nuclear hormone production that is critical in regulating the genes involved in cancer proliferation, metastasis, and survival.^242^ Inhibition of the mevalonate pathway may represent an anticancer strategy. HMGCR, a rate‐limiting enzyme in the mevalonate pathway, has been studied as a therapeutic target for cancer treatment. Statins, HMGCR inhibitors, inhibit cholesterol synthesis and have been commonly used as cholesterol‐metabolism‐targeting drugs in clinical studies for the treatment of cancer patients. Multiple large meta‐analysis studies on statins and cancer mortality found that statin use was associated with significantly decreased all‐cause mortality in cancer patients.^243^ Additionally, numerous clinical studies have reported improved cancer outcomes with the use of statins.^244^ However, in several studies, no beneficial effects were observed in terms of improving long term survival of patients using statins as cancer prevention or as adjuvant therapy.^245^ By contrast, statins can sometimes exhibit a tumor promoting effect,^246^ while in other settings, studies have reported an inhibitory effect of the mevalonate pathway inhibitor on tumor burden.^247^ The evidence previously mentioned suggested that the impact of statins on cancer (inhibiting or promoting) depends on the types of cancer, which may have different biological activities. Kong et al found that imvastatin enhanced the efficacy of enzalutamide‐based therapy, suggesting the therapeutic efficacy of statins to overcome enzalutamide resistance in castration‐resistant prostate cancer. A similar study described a combination therapy approach where docetaxel and atorvastatin improved treatment of prostate cancer. Inhibition of lanosterol synthase, a mevalonate pathway enzyme, was recently reported to suppress cholesterol synthesis and induce cell death of glioma.^248^ Cholesterol metabolism‐related genes appear to be critical to cancer progression and thus represent potential therapeutic targets for cancer treatment.

#### 5.2.2 Blocking cholesterol derivative biosynthesis: Cholesteryl esters and oxysterols

As we have seen, cholesterol esterification plays a role in cancer progression. Avasimibe is a small‐molecule inhibitor targeting ACAT‐1 that inhibits cholesterol esterification and improves the level of intracellular free cholesterol.^249^ Inhibition of cholesterol esterification by avasimibe was found to effectively inhibit the proliferation, metastasis, and invasion of pancreatic cancer.^249^ In addition, elevated free cholesterol may deactivate SREBP1, resulting in the downregulation of the caveolin‐1/MAPK pathway and repression of tumor metastasis and invasion.^249^ Lee et al found that cholesterol esterification inhibition could suppress prostate cancer metastasis and thus represents a potential therapeutic target.^250^ Yang et al confirmed that cholesterol esterification could be inhibited by targeted gene knockout in mouse models or through the use of an ACAT‐1 inhibitor, which stimulated the production of CD3‐TCR clusters on the T cell membrane and thus helping to trigger the proliferation of CD8^+^ T cells.^190^ The production of relevant cytokines and the general cytotoxic antitumor activity were also improved thereby extending the survival period. The accumulated evidence indicates that blocking cholesterol derivative biosynthesis sensitizes cancer cells to other antitumor therapies. A combination of avasimibe with anti‐PD‐1 therapy showed better efficacy than the individual monotherapies in repressing tumor progression.^190^ In addition, avasimibe treatment effectively reduced regulatory T cell populations and enhanced the level of tumor‐infiltrating CD8+ T cell in a lung tumor model, while a combination of avasimibe with a Kras peptide vaccine further increased T cell immunity and improved inhibition of lung tumorigenesis.^251^ CSCs‐DC vaccine combined with avasimibe was shown to have an enhanced therapeutic effect for head and neck cancer in a xenograft model.^252^ In addition, treatment with an ACAT1 inhibitor can improve the antitumor efficacy of anti‐PD1 antibody therapy. The combination of ACAT1 inhibitor with chemotherapy agents such as gemcitabine,^253^ paclitaxel,^254^ or doxorubicin^254^ has also been found to enhance their antitumor effects in pancreatic ductal adenocarcinoma, melanoma, and breast cancer models. Thus, avasimibe can play a role in antitumor and immune enhancement, suggesting that it may be a promising antitumor drug.

27‐HC, a cholesterol hydroxylation metabolite, can promote the extracellular output of cholesterol by activating the LXR signaling pathway.^255^ Recent studies have shown that the level of 27‐HC in breast cancer is significantly higher than that seen in normal breast tissue.^256^ Inhibition of CYP27A1, which is responsible for 27‐HC synthesis, was found to significantly reduce metastasis in animal models of cancer.^257^ This suggests that the development of small‐molecule inhibitors for 27‐HC pathway may reduce the metastasis of tumor cells and represent a new strategy for tumor treatment.

#### 5.2.3 Inducing cholesterol efflux

LXRs are nuclear receptors activated by sterol that regulate the function of a series of target genes related to cholesterol absorption, transportation, outflow, and secretion, and thus play an important role in maintaining the metabolic balance of cholesterol.^258^ Synthetic agonists such as GW3965, TO901317, RGX‐104, and LXR623 have widely been applied in preclinical studies, and have shown promising results for a variety of cancers.^259^, ^260^ Pencheva et al found that expression of ApoE induced by the LXR agonist GW3965 inhibits the invasion, angiogenesis, and metastasis of melanoma.^260^ In addition, TO901317 can degrade melanase through MITF that is induced by Ras and ERK, leading to an inhibition of growth of melanoma cells. In prostate cancer, TO901317 reduced the cholesterol content of lipid raft membranes and phosphorylation of AKT, leading to a signaling disorder and inhibition of the proliferation of prostate cancer cells.^259^ A recent study of renal cell carcinoma suggests that the LXR agonist LXR623 inhibits tumor cells without cytotoxic effects on normal cells. However, a parallel study reported that the cholesterol efflux in stromal cells could also lead to the opposite effect. Ovarian cancer cells can promote membrane‐cholesterol efflux in TAMs that it, in turn, drives TAM‐mediated tumor progression. Genetic deletion of ABC transporters was found to reverse the tumor‐promoting function of TAMs.^199^ The effect of cholesterol efflux and the cross talk between cancer cells and stromal cells requires further investigation.

### 5.3 Targeting phospholipid metabolism

Phospholipids consisting of a phosphoglyceride and sphingolipid are structural molecules of cell membranes with important roles in regulating tumor growth, proliferation, invasion, and metastasis. Targeting the signaling pathways and related rate‐limiting enzymes involved in the biosynthesis of these molecules may represent a strategy for tumor therapy. Anticancer drugs that target phospholipid metabolism are detailed in Table 3.

**TABLE 3:** Summary of anticancer drugs targeting phospholipid metabolism

Target	Compound	Type of cancer	Preclinical model or clinical trial	Refs
PLD	VU0359595	Breast cancer	Xenografts	^328^
	VU‐0155069	Breast cancer	Xenografts	^329^
LPA	Ki16425	Pancreatic cancer	Xenografts	^267^
S1P	FTY720	Lung cancer	Xenografts	^269^
	JTE013	Bladder cancer	Xenografts	^330^
	AB1	Neuroblastoma	Xenografts	^274^
	Sphingomab	RCC	Phase II	^275^
SPHK	SK1‐I	GBM	Xenografts	^331^
	PF543	Colorectal cancer	Xenografts	^332^
	ABC294640	Advanced solid tumors	Phase Ib and II	^272^, ^333^
Ceramide	LCL‑521	Prostate cancer	Xenografts	^273^
	LCL‐385	Prostate cancer	Xenografts	^334^

Abbreviations: LPA, lysophosphatidic acid; PLD, phospholipase D; S1P, sphingosine‐1‐phosphate; SPHK, sphingosine kinase; RCC, renal cell carcinoma; GBM, glioblastoma multiforme

#### 5.3.1 Blocking the phosphoglyceride pathway

Based on our current understanding of potential targets for phosphoglyceride metabolism in cancer, inhibitors of the key enzymes including PLD, PLA1/2, PAP, DGAT1/2, and LPCATs have been explored in a series of studies.^261^, ^262^ In addition to targeting these enzymes, phosphoglyceride derivates and the signaling pathways may also represent potential therapeutic targets. For example, the PI3K/Akt oncogenic pathway is activated in variety of malignant tumors induced by PIP3, and inhibition of the PI3K/Akt pathway is a highly specific molecular target for the development of molecular therapeutics with aberrant PI3K expression.^119^, ^263^ Other metabolites such as AA and eicosanoid involve the COX/LOX, 5‐LOX/LTB4, and PGE2 pathways and are linked to carcinogenesis and the progression of various cancers, suggesting that targeting these pathways may represent a therapeutic strategy.^232^, ^264^, ^265^ Given the role of LPA and LPA receptors in tumor biology, the effective targeting of these receptors has become an important goal in tumor research. This work has largely focused on approaches to reduce LPA synthesis, block LPA metabolic pathway activation, inhibit LPA receptor activity, and block signal transduction in the relevant pathways.^266^ For example, the LPA3 receptor antagonist ki16425 has been reported to inhibit the migration and invasion of pancreatic cancer cells.^267^ Multiple and complex LPA signaling pathways, including ligand‐gated ion channels, RTKs, GPCRs, integrins, and cytokine receptors, may also represent targets that could be even more efficient than targeting LPA or LPA receptor directly.^268^

#### 5.3.2 Blocking the sphingolipid pathway

Because ceramide and sphingosine induce cell cycle arrest and promote cell apoptosis, while S1P promotes cell survival, regulation of the balance between ceramide/sphingosine and S1P has been considered as a new strategy for tumor therapy.^54^ Targeting SPHK‐S1P‐S1PR signaling, including SP1, SPHK1, SPHK2, S1PR1, and S1PR2 by the use of specific inhibitors, represents an innovative strategy for anticancer therapy that has shown efficacy in *in vitro* studies and in tumor xenograft models.^269^ High expression levels of SPHK1 are often associated with the development of resistance to chemotherapy or radiotherapy in colon cancer^270^ and ovarian cancer.^271^ The inhibition of SPHK1 activity can enhance sensitivity of tumor cells to chemoradiotherapy, suggesting the superiority of combination therapy. The SPHK2 inhibitor ABC294640 is currently under investigation in phase Ib and phase II clinical trials for the treatment of patients with solid tumors.^272^ Another strategy induces generation or release of ceramide leading to cancer cell apoptosis. Preclinical studies have identified a series of ceramide inducers and analogs that can accelerate cell death and suppress tumor progression.^273^ FTY720 (Fingolimod), a sphingosine analog derived from myriocin, has widely been used in the treatment of patients relapsing forms of multiple sclerosis as an immunosuppressive agent. Recent studies indicate that FTY 720 can induce apotosis, enhance the effects of chemotherapy, as well as inhibit metastasis therapeutically by targeting sphingolipid signaling in various cancer settings.^54^ FTY720 treatment was found to reduce the incidence of lung cancer caused by urethane, and intraperitoneal injection of SK1‐I enhanced the chemotherapy effect of docetaxel on nonsmall cell lung cancer *in vitro*.^269^ AB1 and JTE‐013 are S1P inhibitors. AB1 shows greater utility than JTE‐013 in blocking S1P‐mediated inhibition of cell migration and antitumor activity in neuroblastomas.^274^ In addition, a clinical trial of the S1P antibody sonepcizumab is underway in renal cell carcinoma. Further investigation of sonepcizumab paired with VEGF‐directed therapies or checkpoint inhibitors represent interesting options for future clinical investigations.^275^

## 6 CONCLUSIONS AND PERSPECTIVES

The development of metabolomics has dramatically expanded our understanding of the metabolic reprogramming that occurs in cancer—something that is now recognized as an important hallmark of tumors.^276^ Metabolomics now represents an important new tool for noninvasive diagnosis and screening for cancer.^277^ Tumors undergo lipid metabolic reprogramming that is strongly linked to malignant transformation and tumor progression. A close association exists between lipid metabolic reprogramming and oncogenic signaling in cancer cells. Oncogenic signal activation can promote expression of lipid metabolism‐associated genes that, in turn, regulates lipid metabolism in cancer cells. Lipid metabolism‐generated intermediates can further enhance the activation of oncogenic pathways in cancer, cooperatively contributing to cancer cell proliferation, invasion, and distant metastasis. In addition to being regulated by oncogenic signaling, lipid metabolism reprogramming in cancer cells can result from signals derived from the TME, such as hypoxia or acidic pH, which can rewire lipid metabolism in cancer cells. This lipid metabolic reprogramming in cancer cells can facilitate their survival in harsh tumor environments. Targeting the genes critical for lipid metabolic reprogramming in cancer may represent an important avenue for cancer therapy. Unfortunately, relatively few compounds targeting enzymes and/or signaling involved in lipid metabolism are currently available for preclinical studies. Statins are widely used drugs for disorders of lipid metabolism that target the rate‐limiting enzyme in cholesterol *de novo* synthesis‐HMGCR. Preclinical studies have identified a positive role for statins in reducing cancer risk, and multiple clinical trials have been carried out to estimate the value of statins as anticancer agents, both in monotherapy, and in combination therapy. To a varying degree of success, the potential mortality benefits of statin consumption has been observed in different types of cancers, including breast cancer, lung cancer, pancreatic cancer, and colorectal cancer.

Apart from cancer cells, cancer‐associated stromal cells also undergo lipid metabolism to support their survival and function in the TME. Lipid metabolism plays a critical role in regulating the function of the noncancer cells within the TME, especially immune‐associated cells. The functional activity of T cells requires SREBP‐mediated upregulation of intrinsic cholesterol biosynthesis, which is also critical for cancer cell survival. Studies are urgently needed to evaluate the potential side effects of drugs targeting SREBP1 on T cell function, and to explore whether there is a reasonable dosage of drug that could achieve therapeutic efficiency with a modest side effect on T cells. Considering the pivotal role of lipid metabolism in immune cell function, some anticancer treatments targeting critical steps in lipid metabolism may also have the side effect of immune suppression‐mediated adaptive drug resistance.

Above all, a comprehensive understanding of how deregulated lipid metabolism regulates cancer progression would be helpful in devising new strategy for the treatment of cancer. It is becoming increasingly apparent that targeting a single molecule or pathway in lipid metabolism is unlikely to result in more effective cancer therapy. The simultaneous inhibition of *de novo* synthesis of lipids and lipid uptake may be needed for optimal treatment. The potential of lipid starvation of tumor cells may be achieved by lipid‐limiting dietary or antiangiogenic therapies that reduce the transportation of lipid into TME. Combinations of drugs that target lipid metabolism together with other antitumor therapies represent more promising strategies to control tumor progression. The discovery of therapeutics strategies interfering with tumor progression and enhancing antitumor immunity is one of the most promising approaches in lipid‐based drug development.
